# Physical activity interventions in adult kidney transplant recipients: an updated systematic review and meta-analysis of randomized controlled trials

**DOI:** 10.1080/0886022X.2025.2480246

**Published:** 2025-03-27

**Authors:** Roseanne E. Billany, Nicolette C. Bishop, Ellen M. Castle, Matthew P. M. Graham-Brown, Sharlene A. Greenwood, Courtney J. Lightfoot, Thomas J. Wilkinson

**Affiliations:** ^a^ Department of Cardiovascular Sciences, University of Leicester, Leicester, UK; ^b^ NIHR Leicester Biomedical Research Centre, Leicester, UK; ^c^ School of Sport, Exercise, and Health Sciences, Loughborough University, Loughborough, UK; ^d^ Faculty of Health Sciences, Curtin School of Allied Health, Curtin University, Perth, Australia; ^e^ Physiotherapy Division, College of Health, Medicine and Life Sciences, Brunel University London, Uxbridge, UK; ^f^ Department of Renal Medicine, King’s College Hospital NHS Trust, London, UK; ^g^ Renal Sciences, Faculty of Life Sciences and Medicine, Kings College London, London, UK; ^h^ Department of Population Health Sciences, University of Leicester, Leicester, UK; ^i^ Leicester Diabetes Centre, University of Leicester, Leicester, UK

**Keywords:** Kidney transplant, exercise, rehabilitation, training, cardiovascular disease

## Abstract

**Background:**

Kidney transplant recipients (KTRs) exhibit a high prevalence of cardiovascular disease (CVD) and adverse changes in physical fitness and body composition. Post-transplant management recommends being physically active and evidence in this field is growing. The aim of this review was to update our previous systematic review and meta-analysis of randomized controlled trials (RCTs) assessing the effects of physical activity and exercise training interventions in KTRs.

**Methods:**

A comprehensive literature search between March 2021 and September 2024 identified seven additional RCTs. Therefore, this updated review and meta-analysis includes 23 RCTs. Outcomes included cardiorespiratory fitness (CRF), strength, blood pressure, body composition, heart rate, markers of dyslipidemia and kidney function, and health-related quality of life.

**Results:**

Twenty-three RCTs, including 1,139 KTRs, were included. The median intervention length was 12 weeks with participants exercising between 2 and 7×/week. Most studies used a mixture of aerobic and resistance training but reporting and intervention content was highly varied. Significant improvements were observed in CRF (V̇O_2peak_; +3.87 mL/kg/min, *p* = .0004), physical function (sit-to-stand-60; +7.72 repetitions, *p* = .0001), and high-density lipoprotein (HDL; +0.13 mmol/L, *p* = .02). Isolated studies reported improvements in strength, bone health, lean mass, and quality of life (QoL). All studies were found to have a high or moderate risk of bias.

**Conclusions:**

Exercise training or increasing physical activity may confer several benefits in adult KTRs, especially through the improvements in CRF and HDL which have been linked to CVD risk. Despite new literature, there is still a need for long-term larger sampled RCTs and more detailed reporting of intervention details and program adherence.

## Introduction

Kidney transplantation is the optimal choice of kidney replacement therapy (KRT) for many patients with end-stage kidney disease (ESKD). It improves patient survival and quality of life (QoL) compared to remaining on dialysis [1]. However, metabolic derangements related to ESKD persist despite transplantation, and cardiovascular disease (CVD) remains a leading cause of morbidity, mortality, and the leading cause of reduced long-term graft survival (16.6%; UK Renal Registry, 2024) [2]. Cardiovascular-related death is the foremost cause of graft loss (40.8%; death with functioning transplant) [3] and clustering of traditional and nontraditional risk factors contribute to this elevated cardiovascular risk [4]. Traditional risk factors include hypertension, diabetes, smoking, obesity, sedentary behavior, and dyslipidemia. Nontraditional (or kidney-specific) risk factors include renal impairment, inflammation, proteinuria, anemia, endothelial dysfunction, and impaired bone mineral metabolism. Strategies that address both traditional and nontraditional risk factors that drive CVD are essential to improve outcomes for this unique patient population.

Appropriate self-management and a healthy lifestyle are recommended for post-transplant care in kidney transplant recipients (KTRs). A key component of this is being sufficiently active (e.g., through structured exercise). In the UK, clinical practice guidelines for exercise in KTRs are closely aligned to those of the World Health Organization for the general population (150 min of moderate to vigorous activity or 75 min of physical activity per week as well as strength exercise) [5]. Despite physical activity levels increasing post-transplant [6,7], less than one-third of KTRs meet these recommendations [8], and levels remain below age-matched healthy controls [6]. Physical limitations [9], comorbidities [9,10], muscle weakness and atrophy [11,12], depression [12], fatigue [9], and fear of injury [13,14] put KTRs at risk of reduced exercise tolerance as well as a lack of guidance [15] and reduced motivation [10,16]. There is strong epidemiological evidence suggesting physical inactivity pre- and post-transplant is associated with increased cardiovascular and all-cause mortality [17–19]; however, programs of exercise, education, and lifestyle have not been embedded into routine clinical care [20]. While safety concerns have been raised, and literature is limited, no significant adverse events have been associated with regular physical activity or exercise training in KTR [21,22].

Our previous systematic review and meta-analysis identified 16 randomized controlled trials (RCTs) which showed beneficial effects of exercise training on cardiorespiratory fitness (CRF), physical function, and high-density lipoprotein (HDL). However, the previous meta-analysis is now three years old and, in a fast-growing field of interest, further RCTs have been published. As part of efforts to update and develop exercise and physical activity clinical practice guidelines for post-transplant care in the UK, using a wider search strategy and an updated methodology, this review was able to include more RCTs and provide a comprehensive meta-analysis and narrative synthesis where appropriate.

Therefore, the overall aim of this review was to update our previous systematic review and meta-analysis of RCTs investigating the effects of physical activity and/or exercise training interventions in KTRs with the following specific objectives.

### Primary

To assess the effects of exercise interventions on ‘hard’ clinical outcomes or events such as mortality, morbidity, hospitalization, and complication rates (e.g., transplant graft function/rejection rates).

### Secondary

- To assess the effects of exercise interventions on other outcomes such as physical fitness (exercise capacity, strength), body composition, cardiovascular risk factors (lipid profile, blood pressure, diabetes), health-related QoL, patient reported outcome measures (e.g., symptoms, fatigue), and markers of kidney function, bone health, and immune function.
- To summarize intervention characteristics based on personnel, setting, frequency, intensity, type, duration, and adherence.

## Materials and methods

A systematic literature search was undertaken per ‘The PRISMA Statement for Reporting Systematic Reviews and Meta-Analyses of Studies That Evaluate Health Care Interventions’ [23].

### Protocol and registration

This review describes an updated review of a previously published systematic review and meta-analysis of Wilkinson et al. [24]. The protocol for this updated review was prospectively registered (original 23 January 2020; re-opened 6 March 2024) on PROSPERO (CRD42020163687).

### Eligibility criteria

#### Types of studies

Randomized clinical trials studying the effect of either physical activity or exercise intervention, either supervised or unsupervised, on outcomes in adult patients with a kidney transplant. Only English language studies were included. Given the high risk of potential selective reporting, unpublished material and abstracts were not included. As specified by the United Kingdom Kidney Association (UKKA) [25], reports detailing protocols, letters, editorials, and conference communications were excluded. Observational studies and interventions consisting only of physical activity counseling were excluded. As this is an updated review, we searched from the last search date in Wilkinson et al. [24] (March 2021) until September 2024.

#### Types of participants

Participants aged ≥18 years who had received a kidney transplant or were awaiting a kidney transplant (including those receiving dialysis therapy) where any intervention commenced post-transplantation. All types of donors were included. Studies conducted in those on dialysis or with non-dialysis CKD were excluded.

#### Types of intervention and comparison

Studies investigating the effects of any form of physical activity and exercise intervention were included. There was no restriction regarding sample size, study location, or duration of the intervention. This review is restricted to studies of a randomized nature with a non-exercise, sham exercise, or guideline-directed care (usual care) control.

#### Types of outcome measures

##### Primary outcomes

As preferred in UKKA clinical guideline recommendation development [25], the primary outcomes of interest were ‘hard’ clinical outcomes or events such as mortality, morbidity, hospitalization, and complication rates (e.g., transplant graft function/rejection rates). These were defined on a per-study basis.

##### Secondary outcomes

- Physical fitness (exercise capacity, strength);
- Body composition and body mass;
- Cardiovascular risk factors (lipid profile, blood pressure, diabetes);
- Health-related QoL outcomes;
- Markers of immune function;
- Markers of bone health;
- Patient reported outcome measures (e.g., symptoms, fatigue);
- Kidney function (creatinine, eGFR);
- Adverse events including serious and non-serious injury.

### Information sources

The following electronic databases were searched from their date of establishment from March 2021 to September 2024: (1) National Centre for Biotechnology Information (NCBI) PubMed (which includes the Medical Literature Analysis and Retrieval System Online (MEDLINE)) and the (2) Cochrane Central Register of Controlled Trials (CENTRAL) (includes Excerpta Medica database (EMBASE), and the WHO International Clinical Trials Registry Platform (ICTRP)).

### Search strategy

**Figure 1.**
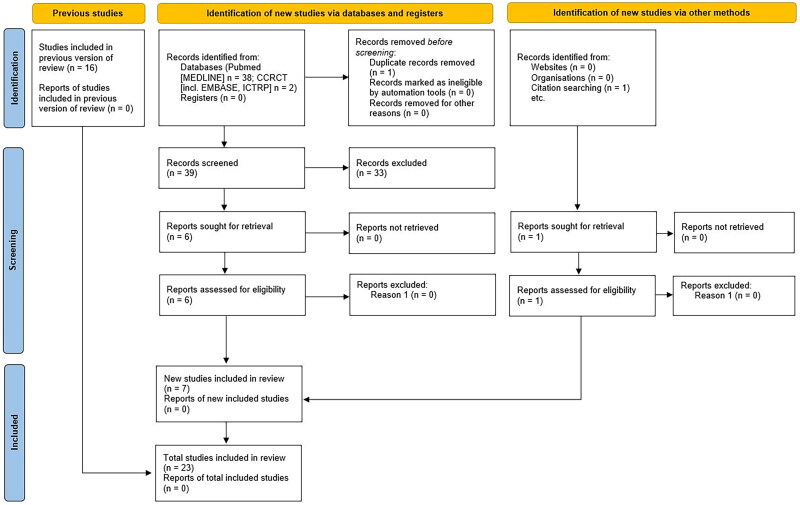
PRISMA flow diagram of a systematic search of the literature and included studies (up to September 2024).

The following MESH search terms were used to search all databases: kidney transplantation; transplant recipients; exercise; exercise therapy; and RCT. Full search strategies can be found in Supplementary material 1. A flow of information through the different phases of the search can be found in Figure 1. The references of recent reviews on exercise and physical activity in KTRs were also hand-searched [26–28].

### Data collection process and data items

Title and abstract screening were performed independently by two independent reviewers (TJW and REB) using Covidence. Initial data extraction was performed by one reviewer (TJW) with extracted data confirmed by NCB, REB, CJL, and SAG. Each full-text article was assessed for risk of bias (RoB) by two authors independently. The data items extracted can be seen in Table 1. Any disagreements were resolved by the inclusion of a third reviewer. Authors were contacted for raw data where data were missing or deemed unusable within the meta-analyses.

**Table 1.** Study characteristics for studies investigating the effects of exercise in kidney transplant recipients.

Study Country	Mean age, years	Transplant vintage	Sample size, *n*^a^	Duration	Exercise intervention summary	Control group (comparator)	Summary of main outcomes (difference from control)	Adverse events
Painter et al. 2003 [29] USA	Ex, 39.7 Con, 43.7	Ex started 1-month post-transplant	Ex, 54 Con, 43	12 months	Home-based aerobic exercise that consisted of walking/cycling 4×/week for at least 30 min (intensity set at 60–65% maximum HR, progressed up to 75–80%)	Usual care group	↑V̇O_2peak_ ↑Lower body strength ↔Body mass ↔BMI ↔Fat mass ↔Lean mass, ↔BMD (total) ↑SF-36 ↔Creatinine ↔BUN ↑Hematocrit ↑Hemoglobin	Not stated
Painter et al. 2003 [30] USA	Ex, 39.7 Con, 43.7	Ex started 1-month post-transplant	Ex, 51 Con, 45	12 months	See above	Usual care group	↔Total cholesterol ↑HDL ↔SBP ↔DPB ↔CVD risk score ↔BMI ↑METS	Not stated
Juskowa et al. 2006 [31] Poland	Ex, 43.8 Con, 46.1	2–3 days post-transplant	Ex, 32 Con, 37	4–5 weeks post-transplant	Supervised every other day for 30 min (on other days unsupervised). Exercise included strengthening the muscles of the upper and lower body as well as abdominal muscles (care of the surgical wound). Breathing, coordination, isometric, and relaxation exercises also included	Standard care control group – standard care not described	↔Hemoglobin ↔Fibrinogen ↔Creatinine ↔Glucose ↔t-Hcy ↔Folate ↔Vit B12 ↔IL-18 ↔Total protein ↔Albumin ↔Total cholesterol ↔LDL ↔Triglycerides ↔HDL	Not stated
Korabiewska et al. 2007 [32] Poland	Ex, 42.6 Con, 43.8	Ex started immediately post-transplant	Ex, 35 Con, 32	12 months	First 6 months involved upper/lower strength training, breathing and coordination exercises for 2/3×/week for 20–35 min. In the second 6 months, walking for 10–20 min 2/3×/week was included. Supervised.	Control group – underwent ‘periodic examinations’	↔Creatinine ↔Hemoglobin ↔Albumin ↔LDH ↔PEF ↔Upper limb strength ↔Joint extension	Not stated
Kouidi et al. 2013 [33] Greece	Ex, 52.1 Con, 52.6	Ex, 22 months Con, 22.1 months	Ex, 12 Con, 12	6 months	Supervised 4×/week for 60–90 min, mainly aerobic-based training (set at 50–75% V˙O_2peak_ or 65–85% maximum HR). AT included interval fitness training (stationary cycling, jogging, step-aerobic exercises, calisthenics, and dancing). RT included strengthening exercises for upper and lower body as well as abdominal muscles. Eight stations (1 set, 12 repetitions at 70% of 1-RM were performed. Increasing to 2–3 sets of 10–12 repetitions at 80% 1-RM.	Sedentary patient control group who were instructed to refrain from exercising during the study period	↔Resting HR ↔SBP ↔DBP ↑Maximum HR ↔V̇Epeak ↑V̇O_2peak_ ↑HRV markers	Not stated
Tzvetanov et al. 2014 [34] USA	Ex, 46 Con, 45	Ex, 8.6 months Con, 10.9 months	Ex, 9 Con, 8	12 months	‘The GH Method’ – consists of individual physical training (one-to-one sessions with a GH coach) using low-impact, low-repetition, resistance-based weight training with two 1-hour sessions each week	Standard of care which includes dietary and exercise counseling by the transplant nutritionist at the time of transplantation and additional dietary and exercise counseling at clinic visits	↔BMI ↔Lean mass ↔Fat mass ↔SBP ↔DBP ↓PWV ↔eGFR ↔Creatinine ↔Lipoproteins ↔Triglycerides ↔Fasting blood glucose ↔Hemoglobin ↑SF-36	Not stated
Riess et al. 2014 [35] Canada	Ex, 56.9 Con, 52.4	Ex, 6.4 years Con, 9.1 years	Ex, 16 Con, 15	12 weeks	Aerobic training 3×/week at 60–80% V̇O_2_max for 30–60 min on bike or treadmill. Strength 2×/week. Only lower body exercises included. Fifty percent 1-RM for 2 sets of 10–15 repetitions. The intensity increased by 5–10% when 2 sets of 15 repetitions were performed well. Leg press, leg extension, and leg curl. Supervised	Not provided with exercise guidelines and continued with their usual activities	↑V̇O_2peak_ ↔Watts ↑Cardiac output ↑Maximum HR ↔Stroke volume ↔Arterial compliance ↔SBP ↔DBP ↑Lower body strength ↔Lean mass ↔CVD risk score ↑SF-36	Not stated
Pooranfar et al. 2014 [36] Iran	Only total reported: 36.3 years	Transplantation 2–3 years prior to study	Ex, 29 Con, 15	10 weeks	60–90 min session, 3×/week. Combination of aerobic cycling, treadmill (set at 40–70% maximum HR) and free weights using a circuit format 45–65% of maximum frequency.	‘The control group experienced no regular physical exercise’	↑Sleep quantity ↑Sleep quality ↓LDL ↔HDL ↓Total cholesterol ↓Triglycerides	Not stated
Greenwood et al. 2015 [37] UK	Ex^b^, 54.3 Con, 49.5	Ex^b^, 29.3 weeks Con, 27.7 weeks	Ex^b^, 40 Con, 20	12 weeks	Aerobic (AT) or resistance training (RT) groups 3×/week. AT consisted of stationary bike, treadmill and elliptical trainer, 80% HR maximum, RPE 13–15. Training duration was progressed to 60 min per session. RT intensity set at 80% 1-RM for both upper and lower body exercises. Frequency built to 3 sets of 8–10 repetitions (bench press, latissimus pulldown, bicep curl, triceps pull down, leg press, knee extension, hamstring curl, and calf raises). AT group were provided with a HR monitor and the RT were provided with Therabands, ankle weights and free weights.	Seen in routine clinic and not referred to any exercise intervention pathways	↑PWV ↑V̇O_2peak_ ↑Lower limb strength (RT group only) ↑STS-60 (RT group only) ↑DASI ↔SF-36 ↔eGFR ↔BMI ↑Weight ↓Resting HR (RT group only) ↔SBP ↓DBP (RT group only) ↔CRP ↔IL-6 ↔TNF-a ↔TNFR-1 ↔TNFR-2 ↔Fetuin	No adverse events observed
Karelis et al. 2016 [38] Canada	Ex, 45.3 Con, 39.4	Ex started 6–8 weeks post-transplant	Ex, 12 Con, 12	16 weeks	Supervised resistance training 3×/week. 1× supervised, 2× home. Sessions lasted between 45–60 min. Supervised sessions included (1) leg press; (2) chest press; (3) lateral pull downs; (4) shoulder press; (5) arm curls; (6) triceps extensions; and (7) sit-ups at 80% 1-RM. Three sets of 10 repetitions. Resistance was provided with elastic bands for 4 exercises (seated row, lying chest press, overhead triceps extension, standing biceps curls) and the patient’s own body weight for 3 exercises (squats, lunges, push-ups).	Control group – instructed not to perform any structured exercises	↑WHO-5 well-being score ↔Body mass ↔Fat mass ↔Lean mass ↔V̇O_2peak_ ↔Strength ↔Triglycerides ↔Total cholesterol ↔HDL ↔LDL ↔Glucose ↔A1C ↔HOMA	No injuries or adverse events observed
Eatemadololama et al. 2017 [39] Iran	Ex, 27.4 Con, 36.0	Transplant <1 year prior	Ex, 12 Con, 12	12 weeks	Supervised 2×/week. 10 min stretch, 10 min walking on treadmill, 20 min cycling, 20 min upper RT and 20 min lower RT, 10 min cool down. 1 set of 10–15 repetitions at 50% 1RM. Resistance increasing 5–10%.	‘Control group without any intervention’	↑BMD (femur) ↔BMD (lumbar spine)	Not stated
Onofre et al. 2017 [40] Brazil	Ex, 37.0 Con, 35.6	Exercise began 1-day post-operative	Ex, 30 Con, 33	Until discharge (mean in Ex group was 7.1 days)	Daily post-operative supervised physiotherapy for 30 min (upper limb movement, walking, step-ups). Resistance training introduced from day 2 – shoulder diagonals and elbow flexions 3 sets of 10 reps with load determined by tolerance (avg. 2 kg for women and 4 kg for men). Rest of 2 min between sets. Progressive each day	Standard care (including daily visits from physio and mobility encouragement)	↔6MWT ↑Respiratory muscle strength ↑Maximum expiratory pressure ↔Upper limb strength ↔Lower limb strength	Not stated
O’Connor et al. 2017 [41] UK	Ex^b^, 54.3 Con, 49.5	Ex^b^, 29.3 weeks Con, 27.7 weeks	Ex^b^, 40 Con, 20	9-month self-managed physical activity	Encouraged to engage with community exercise pathways following end of involvement in 12-week exercise programme (see Greenwood et al. [37])	Usual care (no specific exercise guidance other than at routine appointments)	↓PWV ↔V̇O_2peak_ ↔SBP ↔DBP	See footnote^c^
Kumar et al. 2020 [42] India	Ex, 36.2 Con, 35.1	Unclear	Ex, 61 Con, 61	12 weeks	Supervised graded aerobic and upper and lower body resistance exercise over 3 phases, 2×/week supervised, 2×/week at home Phase I: graded gravitational stress and limb leverage increased for strengthening, RPE based ambulation in progression (6–11); resistance up to 50% of 10 RM Phase II: Graded aerobic exercises (walking/treadmill walk/bicycle ergometer training); resisted exercises − 50–65 of 10 RM% flexibility exercises; RPE-guided progression in exercise volume (RPE: 9–14) Phase III: Progressive, graded aerobic exercises (walking/treadmill walk/bicycle ergometer training); resisted exercises: 65–85 of 10 RM% flexibility exercises; RPE-guided progression in exercise volume (RPE: 9–14)	Control group received routine care, including chest physiotherapy, breathing exercises, and graded ambulation	↑6MWT ↑Lower limb strength ↓Fatigue	Not stated
Hernández Sánchez et al. 2021 [43] Spain	Ex, 49.7 Con, 48.6	Ex, 115 months Con, 88 months	Ex, 8 Con, 8	10 weeks	Supervised exercise 2×/week, 60 min sessions. Upper and lower body resistance training including leg press, rowing pulley, leg curl, fly machine, machine calf raises, knee extension, and core work, 3 sets of 10 repetitions at 10-RM.	No information stated	↑SF-36 ↔KDQOL-SF ↔Lower limb strength ↑6MWT ↑HGS ↑STS-60 ↑TUAG ↔Muscle size (thickness)	None observed
O’Brien et al. 2020 [44] USA	Ex, 65.7 Con, 65.1	Ex, 7.0 years Con, 7.2 years	Ex, 27 Con, 26	6 months	Intervention group received group sessions involving the SystemCHANGE™ approach plus a activity tracker (FitBit). This 4-step approach encourages individuals to adopt changes in behavior to increase physical activity. Progress was reviewed each month.	Control group received the activity tracker but no additional information other than educational information regarding self-care after transplantation	↑Daily steps ↔SPB and DBP ↓Resting HR ↔6MWT ↔Body mass ↔BMI ↔Waist circumference	Not stated
O’Brien et al. 2021 [45] USA	Ex, 65.7 Con, 65.1	Ex, 7.0 years Con, 7.2 years	Ex, 27 Con, 26	6 months	Self-directed exercise with activity tracker following completion of a 6-month programme (see O’Brien et al. [44])	Continued as above	↔Daily steps ↔SPB and DBP ↔Resting HR ↔Body mass ↔BMI ↔Waist circumference	Not stated
Lima et al. 2021 [46] Brazil	Ex, 54 Con, 43	Ex, 4 ± 1 years Con, 4 ± 2	Ex, 7 Con, 5	12 weeks	3×/week Session: 5-minute warm-up, 30 min of aerobic cycling (60–80% of heart rate reserve), and resistance exercise. Resistance exercise: 2 sets of 5 reps with 60 s rest of the following: dumbbell bent row, dumbbell bench press, dumbbell overhead press, dumbbell squat, and standing knee flexion (intensity: 6–7 on the OMNIRES scale)	Typical post-transplant care	↔Body mass ↔BMI ↔BMD (total) ↓% Body fat ↓Fat free mass ↑HGS ↑V̇O_2peak_ ↓Creatinine ↑eGFR	Not stated
Kastelz et al. 2021 [47] USA	Ex, 47.29 Con, 43.18	Unclear	Ex, 80 Con, 33	12 months	2×/week 60 min supervised resistance upper body: biceps curl, chest press, shoulder press, triceps extension/pushdown, lat pull-downs, front row, sit-ups; lower body: leg extension, leg curl, leg press, leg abduction, leg adduction. Exercise was prescribed in 3 phases	Standard care	↑Global physical health ↑Global mental health ↑Physical function ↔Anxiety ↔Depression ↔Sleep disturbance	Not stated
Michou et al. 2022 [48] Greece	Ex, 52.9 Con, 53.0	Ex, 47.4 ± 18.3 months Con, 47.8 ± 18.1 months	Ex, 10 Con, 11	6 months	3×/week, 60–90 min, home-based (three familiarization sessions prior) Aerobic (stairs or stationary bike) and resistance training Aerobic starts 15 min and increases to 40 min (60–80% max HR) Resistance: 6 exercises, 2 sets, 8–10 reps. Body weight and then 1 kg dumbbells. E.g., Shoulder press, biceps curl and triceps extension, leg flexion and extension	Standard care and maintaining current lifestyle	↓Glucose ↔Triglycerides ↑HDL ↔LDL ↔HbA1c ↑V̇O_2peak_	None observed
Hemmati et al. 2022 [49] Iran	Ex, 32.9 Con, 37.8	Transplantation 1–3 years prior to study	Ex, 13 Con, 10	10 weeks	3× week, supervised 60–90 min 15–20 min aerobic bike/treadmill (40–70% HR max) Resistance training using body weight or free weights at 45–65% of max frequency	Usual care	↔IL-4 ↓IL-6 ↔IL-31 ↔IL-35 ↓TNF-a ↔Weight ↔BMI	Not stated
Zhang et al. 2023 [50] China	Ex, 43.16 Con, 42.06	Ex started immediate post-transplant	Ex, 54 Con, 54	3 months Then further 3 months before follow- up	Nurse led programme. Pre- and post-discharge. Non-ambulatory to ambulatory (after passing TUAG test). Daily combined aerobic and resistance training. Once discharged participants sent daily video to nurse. Dumbbells for resistance training	Routine nursing care (including exercise suggestions)	↓Fatigue ↔HR ↔BMI ↔Glucose ↔Creatinine ↔Triglycerides ↑6MWT ↔SF-36 ↓Anxiety ↓Depression ↔% Body fat ↔Fat free mass	None observed
Knobbe et al. 2024 [51] Netherlands	Ex 53.9 Con, 51.9	Ex, 5.5 (3.3–9.7) months Con, 5.9 (3.8–8.6)	Ex, 77^d^ Con, 74^d^	15 months (3 months structured supervised exercise − 12 months lifestyle counseling)	3 months 2× week supervised exercise (30 min dynamic resistance training, 30 min aerobic training, 30 min supervised sports) Aerobic cycling guided by Watts achieved in maximal exercise test (progressive increase from 50–80% of maximum Watts). Target heart rate for walking matched with heart rate during cycling Resistance training guided by maximal strength testing Lifestyle counseling (five sessions in total at 6 weeks, 12 weeks, 16 weeks, 6 months, and 15 months.	Standard post-transplant care according to local protocol	At 15 months ↔SF-36 domain physical functioning ↔SF-36 physical and mental component scores ↔EQ5D ↑Muscle strength, *Z* score ↑V̇O_2peak_ ↑Peak power output ↔Daily steps ↔Physical activity ↔Weight ↔BMI ↔% Body fat ↓Waist size ↑HDL ↔SBP and DBP ↔LDL ↔Glucose	Fully reported Analyses of safety outcomes showed no safety concerns

Ex: exercise group; Con: control; 6MWT: 6-min walk test; PWV: pulse wave velocity; SBP: systolic blood pressure; DBP: diastolic blood pressure; HR: heart rate; 1-RM: 1-repeitition maximum; STS-60: sit-to-stand-60 s test; HGS: handgrip strength; TUAG: timed-up-and-go; DASI: Duke Activity Status Index; eGFR: estimated glomerular filtration rate; BMI: body mass index; CRP: C-reactive protein; IL-#: interleukin-#; TNF: tumor necrosis factor (α, alpha, R, receptor); SF-36: Short-form 36 questionnaire; KDQOL-SF: kidney disease quality of life-short form; CVD: cardiovascular disease; VE: ventilation equivalent; HRV: heart rate variability; METS: metabolic equivalent; A1C: glycated hemoglobin; HOMA: homeostatic model assessment; LDH: lactate dehydrogenase; PEF: peak expiratory flow; BMD: bone mineral density; ↑: significantly higher; ↔: no significant difference; ↓: significantly lower.

^a^ Total sample size of those randomized.

^b^ Combined age and transplant durations of the exercise groups as was included in the meta-analysis.

^c^ 15.4% patients hospitalized in Ex group, compared to 40% in control group (from baseline). No difference in rejection rates between groups, no deaths. Higher incidence (30.8%) in Ex groups of NODAT compared to 10% in control.

^d^ Data for third group (exercise + diet intervention) excluded.

### Risk of bias in individual studies and publication bias

Pairs of reviewers working independently assessed the RoB for each study. The RoB was assessed according to the Cochrane Risk of Bias assessment tool and defined as high, low, or unclear across the five domains [52]. Overall study risk was determined as (i) low RoB (all criteria graded low), (ii) moderate RoB (one criterion graded high or two unclear); and (iii) high RoB (more than one criterion graded high or more than two unclear). There was an agreement in 64% of statements (*κ* = 0.37). Any differences and discrepancies were reviewed by TJW (as a third reviewer). Funnel plots were used to assess the risk of publication bias if there were >10 studies.

### Summary measures

All outcomes were treated as continuous data and interpreted as mean differences. Analyses were primarily based on final values post-intervention. Where baseline imbalances existed between groups, analyses were based on changes from baseline [53]. Where appropriate, post-intervention values were calculated from available data [54]. Where studies had more than one relevant intervention group, means and standard deviation (SD) were combined using the methods described within the Cochrane Handbook [54].

### Synthesis of results

#### Meta-analysis

Statistical heterogeneity was assessed using the *I*^2^ test [53]. Although an *I*^2^ value ≥40% is conventionally used to indicate moderate heterogeneity in the literature, we also took into account the magnitude of effects and strength of evidence (e.g., *p* value from *χ*^2^ test) in our interpretation [55]. When considerable heterogeneity (>50%) was identified, efforts were made to reduce the *I*^2^ value by removing studies at high RoB (studies with ≥2 criteria graded high). If *I*^2^ was reduced then both forest plots are displayed (both with and without studies removed). If the *I*^2^ value remained considerable then a meta-analysis was not performed and outcomes were narratively described (as described below). Forest plots of excluded meta-analyses are provided in Supplementary material 2. With an overall intention to generalize the results beyond the included studies [55], and as the effects of exercise interventions were deemed to be highly variable according to age, sex, training duration, frequency, and type characteristics, a random-effects model was chosen to calculate the average distribution of treatment effects that can be expected. When the standardized mean difference (SMD) was used, we translated this to the units of the test to make it more clinically relevant by multiplying the SMD generated from the meta-analysis by the pooled post-intervention SDs [56].

Data analysis was conducted using Review Manager (RevMan) [Computer program]. Version 5.4.1, The Cochrane Collaboration, 2020.

#### Narrative synthesis

When meta-analysis was not appropriate, data were narratively described within the text (if two or more studies reported on the outcome) and a direction of effect was generated for all outcomes (in Table 1). An estimate of the proportion of effects favoring the intervention (exercise) was calculated along with a 95% confidence interval. To assess if there was any evidence of an effect of exercise, summary outcome metrics are shown using a vote count of each effect direction as recommended by the Cochrane Handbook. A sign test was used to examine the probability of observing the given pattern of positive effect direction across studies if the null hypothesis of even distribution of positive and negative results was true. Statistical significance was recognized as *p* < .05.

## Results

### Study selection

Following a re-run of searches in September 2024, seven new studies were identified [45–51] and as such, in total, 23 studies were eligible for inclusion. Due to inadequate reporting and a wide heterogeneity of measures, 19 of these trials provided information for use in meta-analyses. Figure 1 provides a PRISMA diagram of the included studies.

We found three instances of reports from the same original study. O’Connor et al. [41] was a 12-month follow-up of Greenwood et al. [37]. Both studies were retained as they provided unique data on the effect of exercise and the longer-term effect of self-managed physical activity. Two studies by Painter et al. [29,30] were identified (an initial study and a secondary analysis). Both reports were retained as they reported different data and patient sample sizes and may be prone to bias. A 6-month follow-up by O’Brien et al. [45] was found and included in the qualitative analysis as it provided information on intervention maintenance. The three follow-on/secondary reports were not included in the sample size descriptions below.

### Study characteristics

Table 1 provides a summary of the characteristics of the included trials.

In total, *n* = 1,139 patients were randomized to receive exercise (*n* = 618) or control (*n* = 521) across the 20 unique studies. Total study sample sizes ranged from *n* = 16 to *n* = 221. The median and mean total sample sizes were *n* = 57 and *n* = 49, respectively.

Included trials were published between 2002 and 2024 in English and were conducted in Brazil (2), UK (2), USA (6), China (1) Canada (2), Greece (2), Poland (2), Spain (1), Netherlands (1), India (1), and Iran (3). When stated, all studies utilized a 1:1 or 1:1:1 randomization, apart from Kastelz et al. (2:1) [47].

Nineteen studies reported two distinct groups (an exercise group and a control group) while the ExeRT study [37,41] had three groups (two exercise arms – separated into resistance training (RT) and aerobic training (AT)). Data from a third non-kidney transplant ‘healthy’ group in the study by Kouidi et al. were excluded [33], and data from a third combined exercise and diet intervention group in the study by Knobbe et al. were excluded [51]. In the latter study, the 15 month intervention consisted of 3 months of structured exercise training combine with lifestyle counseling and a further 12 months of lifestyle counseling; included meta-analyzed data is at 15 months [51].

The control group was often described as a ‘usual/standard care’ group. Two studies actively instructed patients in this group not to exercise [33,38]. The ‘standard care’ in one study involving patients in acute post-transplant aftercare involved daily physiotherapist visits and mobility encouragement [40]. In Kumar et al., control participants received basic physiotherapy [42], while in both of the O’Brien et al. reports [44,45], the control group received an activity tracker but no supplementary behavior change intervention. The usual care group in Zhang et al. involved routine nursing care that consisted of exercise suggestions [50].

All exercise interventions were conducted post-transplantation. Four studies recruited patients immediately or recently post-transplant [29,31,32,38]. Two studies completed baseline assessments before transplantation [40,50]. One study employed immediate daily post-operative supervised physiotherapy for 30 min with RT introduced from day two; this was continued until discharge [40]. Juskowa et al. recruited patients 2–3 days after transplantation [31] and participants in Painter et al. were recruited <1 month after transplantation [29]. Participants in Zhang et al. commenced exercise on post-operative day 3 after a safety assessment [50]. Three studies [37,39,49] recruited participants during the first year post-transplantation.

### Reporting of exercise interventions

Detailed reporting of the exercise interventions was generally lacking in several studies and the details provided ranged widely (Table 2).

**Table 2.** Summary of the reporting of exercise intervention characteristics.

Study Country	Personnel	Setting	Frequency	Duration	Type	Intensity – aerobic	Intensity – resistance	Additional contact	Adherence/compliance
Painter et al. 2002 [29] USA and Painter et al. 2003 [30] USA	Exercise study staff	Home-based	Min 4×/week	Programme: 12 months Sessions: Working up to min 30 min/session	Aerobic (primarily walking or cycling)	Initially 60–65% of max heart rate; gradually increased to 75–80% (approx. every 2 weeks increment)	NA	Telephone contact weekly, reducing to every other week	67% of intervention participants were regularly active at 12 months compared to 36% of the control
Juskowa et al. 2006 [31] Poland	Physio-/physical-therapist	During hospitalization for transplant	Everyday (one day supervised; one day solo)	Programme: Until hospital discharge Sessions: 30 min/session	Exercises strengthening the muscles of the upper and lower limbs; exercises strengthening the abdominal muscles (care of the surgical wound); and breathing, coordination, isometric, and relaxation exercises	Not reported	Not reported	NA	Not reported
Korabiewska et al. 2007 [32] Poland	Not reported	During hospitalization for transplant and following (location not stated)	First 6 months: 2–3×/week 6 months+: as above + intensive walks every 2–3 days	Programme: During hospitalization and for 1 year after Sessions: First 6 months: 20–35 min 6 months+: as above + intensive walks every 2–3 days; 10–20 min	Strengthening of upper and lower extremity muscles, stomach muscles, breathing exercises, coordination, and isometric and relaxation exercises Walking	Not reported	Not reported	Not reported	Not reported
Kouidi et al. 2013 [33] Greece	Exercise trainers specialized in physical rehabilitation	In-center	4×/week	Programme: 6 months Sessions: 60–90 min (gradual increases) *10 min warm-up* *30*–*40 min aerobic* *10*–*30 min strength* *10 min cool down*	Aerobic: interval fitness training (stationary cycling, jogging, step-aerobic exercises, calisthenics and dancing) Resistance: 8 stations for abdominal, upper and lower limbs	Close to the anaerobic or ventilatory threshold (50–75% V̇O_2_peak or 65–85% HRmax)	1 set,12 reps at 70% of 1 RM with a target of 2–3 sets of 10–12 reps at 80% of 1 RM	NA	Patients included in analysis if 80% of sessions attended (all but 1 achieved this)
Tzvetanov et al. 2014 [34] USA	Coach	In-center	2×/week	Programme: 12 months Sessions: 1 h	Low impact, low-repetition, resistance-based weight training	NA	Not reported	NA	100%
Riess et al. 2014 [35] Canada	Exercise physiologists	In-center	5×/week Aerobic 3×/week Resistance 2×/week	Programme: 12 weeks Sessions: aerobic: 30–60 min Resistance: not reported	Aerobic: cycle ergometer and treadmill Resistance: not reported	60–80% VO_2_peak with increases when RPE <11 or if HR ≤60% V̇O_2_peak	50% 1RM for 2 sets of 10–15 reps increasing by 5–10% when achieved with good technique	NA	Participants attended 81% ± 31% of required exercise sessions at 85% ± 1% of baseline peak HR. Strength training was performed for 2 sets of 10 repetitions at 61% ± 7% of baseline 1RM
Pooranfar et al. 2014 [36] Iran	Researcher/sports physiologist	In-center	3×/week	Programme: 10 weeks Sessions: 60–90 min *10*–*15 min pre-warming* *10 min cool down* *9–17 stations, 3–6 circles in each session, 1–2 min between each station and 3–5-min between circles*	Aerobic: ergometer bicycle, treadmill Resistance: free weights	40–70% maximum HR intensity	45–65% of max frequency	NA	Not reported
Greenwood et al. 2015 [37] UK and O’Connor et al. 2017 [41] UK	Physio-/physical-therapist	Home-based/in-center mix	3×/week *2*×/*week in-center* *1*×/*week home-based*	Programme: 12 weeks Sessions: aerobic: 2 × 30 min, progressing to 1 × 60 min Resistance: 60 min (or exercise completion) *Min 5 min warm-up and cool-down*	Aerobic: In-center: recumbent stationary exercise cycles, a treadmill, and elliptical trainer Resistance: bench press, latissimus pulldown, bicep curl, triceps pull down, leg press, knee extension, hamstring curl, and calf raises	Aerobic group only: In-center: 80% HR reserve Home: 13–15 RPE	Resistance group only: 1–2 sets of 10 reps at 80% 1RM. Progression to 3 sets of 8–10 reps	Weekly telephone calls	26 participants completed the exercise interventions (23% attrition) with 87.4% of 36 sessions completed Aerobic group; all participants could complete a 60-minute session Resistance training group; all participants could complete three sets of 10 repetitions as per protocol
Karelis et al. 2016 [38] Canada	Kinesiology students	Home-based/in-center mix	3×/week *1*×/*week in-center* *2*×/*week home-based*	Programme: 16 weeks Sessions: 45–60 min *10 min warm-up*	Leg press; chest press; lateral pull downs; shoulder press; arm curls; triceps extensions and sit-ups	NA	In-center: 3 sets of 10 reps at 80% 1RM; load increased gradually based on 1RM change; 1–1.5 min rest between sets Home: resistance was provided with elastic bands for 4 exercises (seated row, lying chest press, overhead triceps extension, standing biceps curls) and body weight for 3 exercises (squats, lunges, push-ups)	Not reported	Compliance with the exercise sessions was 80%; 2 drop outs in the exercise group
Eatemadololama et al. 2017 [39] Iran	Exercise specialists	In-center	2×/week	Programme: 12 weeks Sessions: 80 min *10 min stretching; 10* *min walking on treadmill; 10 min cycling; 20 min* *upper body resistance training; 20 min lower body resistance training; 10 min cool-down*	Aerobic: walking on treadmill, cycling Resistance: not reported	Not reported	10–15 reps at 50% 1 RM increasing 5–10%	NA	Not reported
Onofre et al. 2017 [40] Brazil	Physio-/physical-therapist	During hospitalization for transplant	Everyday	Programme: until post-operative discharge Sessions: 30 min	Breathing exercise, walking, step exercise	Day 1+: 4× laps of 30 m corridor Day 2+: increase 1 lap per day	Day 1+: 5 reps of step exercises on 25 cm step Day 2+: 3 sets of 10 reps; weight determined by tolerance; 2 reps of up and down 12-step flight of stairs Day 3+: increase stair climb 1 flight per day	NA	Not reported
Kumar et al. 2020 [42] India	Physio-/physical-therapist	Home-based/in-center mix Phase I – up until discharge Phase II (3–6 weeks) – gradual home activities Phase III (6–12 weeks) – home-based activities	4×/week 2× supervised; 2× home-based	Programme: 12 weeks Sessions: not reported; gradual volume increase	Graded ambulation, body weight exercise, walking, treadmill, bicycle	Phase I: RPE 6–11 Phase II and III: RPE 9–14	Phase I: 50% of 10 RM Phase II: 50–65% of 10 RM Phase III: 65–85% of 10 RM	Phone call reminders	Not reported
Hernández Sánchez et al. 2021 [43] Spain	Exercise physiologists	In-center	2×/week	Programme: 10 weeks Sessions: ∼60 min *Warm-up 7 min walking + specific joint mobility*	Resistance machines and bodyweight leg press, rowing pulley, leg curl, fly, calf raises, knee extension, and core work	NA	3/4 sets of 10 reps at 10 RM with 1 min rest between sets	NA	100% of total number of planned sessions
O’Brien et al. 2020 [44] USA and O’Brien et al. 2021 [45] USA	Research team	Home-based/in-center mix *6*× *monthly group sessions*	Intervention to increase overall steps walked – therefore frequency not reported	Programme: 6 months Sessions: NA	Walking/steps	NA	NA	‘Ongoing contact’	Participants wore their activity trackers consistently and implemented their daily step goal
Lima et al. 2021 [46] Brazil	Not reported	In-center	3×/week	Programme: 12 weeks Sessions: not reported *5 min warm-up, 30 min aerobic, resistance training, cool down, stretching*	Aerobic: Resistance: free weights; dumbbell bent row, dumbbell bench press, dumbbell overhead press, dumbbell squat, and standing knee flexion	60–80% HR reserve	2 sets of 15 reps, 60 s rest between sets; 6–7 on the OMNIRES scale	NA	Not reported
Kastelz et al. 2021 [47] USA	‘Trained personnel’	In-center	2×/week	Programme: 12 months Sessions: 60 min *5*–*10 min light warm-up + stretching included*	Biceps curl, chest press, shoulder press, triceps extension/pushdown, lat pull-downs, front row, sit-ups; lower body: leg extension, leg curl, leg press, leg abduction, leg adduction	NA	Phase I (2–4 weeks): set 1, 30 s of exercise using 2 s concentric and 3 s eccentric contraction; set 2, as above. 3 or below on 10-point fatigue scale Phase II (8–10 weeks): set 1, 15–20 reps 3 or below on 10-point fatigue scale; set 2, 10–12 reps on day 1 at perceived exertion of 3–4, 6–8 reps on day 2 of each week, a perceived exertion of 4–5 Phase III: set 1, 25–30 reps at a perceived exertion of 4–5; set 2, 12–15 reps at a perceived exertion of 4–6. All exercises were done without going above 4 on the Fatigue Scale	NA	Adherence was 73% in the exercise group (75% when including the 2 participants that had a job prior to enrolment)
Michou et al. 2022 [48] Greece	Researchers	Home-based *Three in-center familiarization sessions in week 1*	3×/week	Programme: 6 months Sessions: 60–90 min Aerobic: initially 15 min increasing by 5 min every 2 weeks *10 min warm-up; 10 min recovery stretching*	Aerobic: stationary bike, stair climbing Resistance: shoulder press, bicep curl, triceps extension, leg-flexion	60–80% of the max HR	2 sets of 8–10 reps initially body weight, progressing to rubber bands, balls and dumbbells (1 kg)	Weekly phone calls and monthly home visits	Three patients withdrew from exercise group
Hemmati et al. 2022 [49] Iran	Researchers/clinical exercise physiologists	In-center	3×/week	Programme: 12 weeks Sessions: 60–90 min *10 min warm-up, 15*–*20 min aerobic exercise, resistance exercise, 10 min cool down*	Aerobic: cycle ergometer, treadmill Resistance: free weights and body weight	40–70% max HR progressing to 65–85%	45–65% max frequency	NA	Not reported
Zhang et al. 2023 [50] China	Nurses	Ward/home-based post-discharge Non-ambulatory phase (post-op day 3 until TUAG <20 s) and ambulatory phase	Every day (2×/day)	Programme: 3 months + 3 months follow-up Sessions: various from 2 min to 25 min	Aerobic: walking, Baduanjin Resistance: dumbbells and resistance bands	Not reported	Increasing reps first, then sets, then resistance Increase from 1–2 sets to 3–4 sets of 8–12 reps	Telephone reminders Participants sent daily videos to nurses	Considered drop out if 3 consecutive sessions missed The adherence rate in the intervention group was 98.11%
Knobbe et al. 2024 [51] Netherlands	Physio-/physical-therapist	In-center (for 3 months)	2×/week	Programme: 3 months exercise + lifestyle counseling (5 sessions across 15 months) Session: 90 min *30 min dynamic resistance training, 30 min aerobic training, 30 min supervised sports*	Aerobic: cycling Resistance: dynamic of all major muscle groups Sports activities: swimming or playing hockey/soccer	10 min cycling at 50% of Watt max and 10 min walking at the same HR as cycling progressing to 14 min cycling at 80% of Watt max and 14 min walking at the same HR as cycling	1 set of 30 reps (25% max strength) with gradual progression to 3 sets of 10 reps (60% max strength)	NA	44 participants (17 control, 9 exercise intervention and 18 exercise plus diet intervention) did not adhere to or complete the allocated treatment

1-RM: 1-repeitition maximum; HR: heart rate; RPE: rating of perceived exertion; TUAG: timed-up-and-go.

### Risk of bias within studies

**Figure 2.**
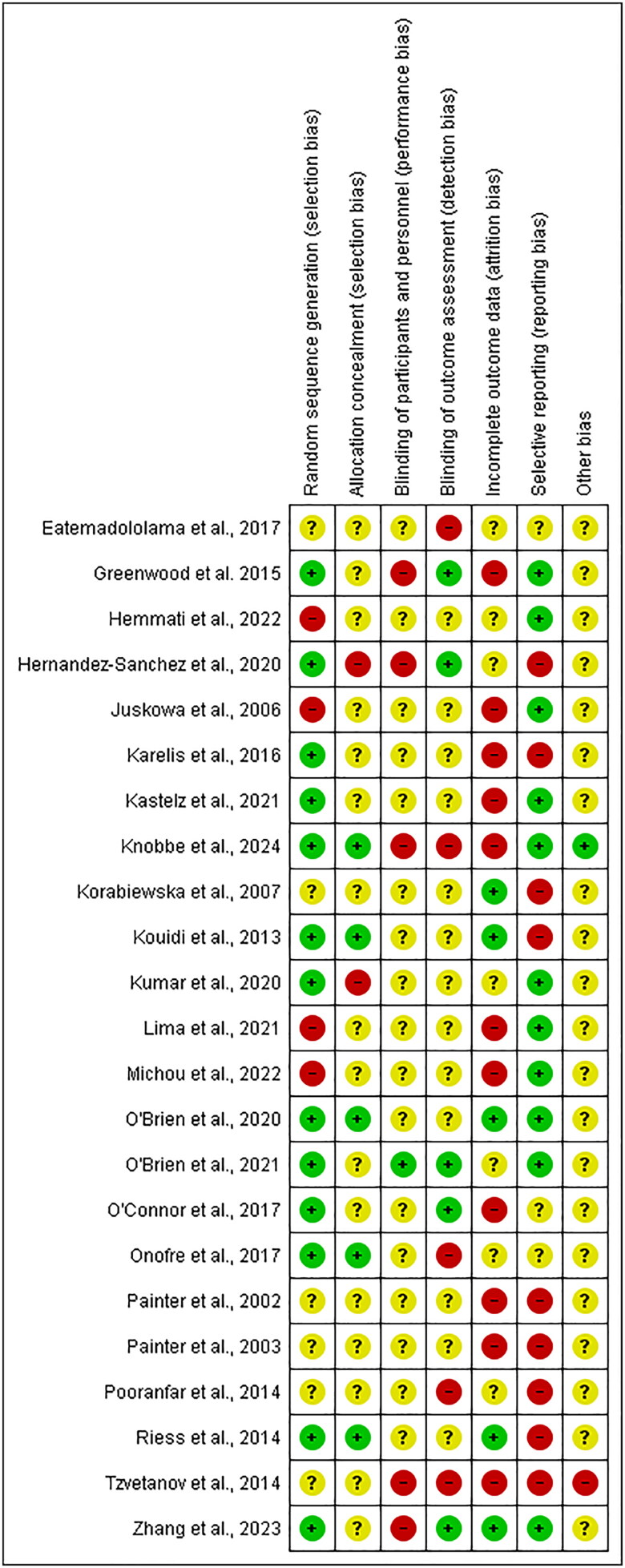
Risk of bias per study.

**Figure 3.**
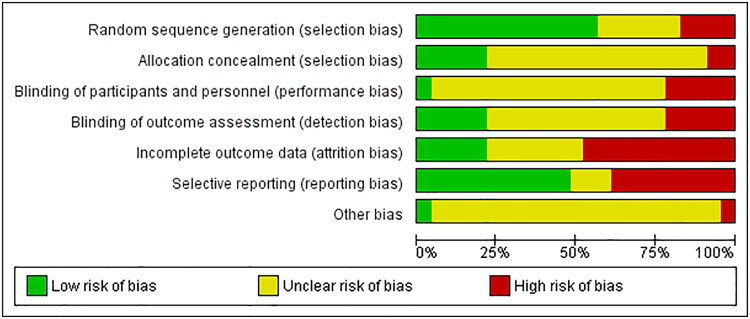
Risk of bias summary.

The majority of studies (19/23) were found to be at high RoB, four were of moderate RoB, and zero at low RoB. Selective reporting and incomplete outcome data were the most frequent causes of bias identified. Randomization processes were generally well described across most studies. The RoB per study and a summary of the RoB can be found in Figures 2 and 3, respectively.

### Narrative synthesis

No studies reported data on mortality, morbidity, or hospitalization.

#### Body composition

##### Markers of lean mass and muscle mass or size

Eight studies reported changes in markers of either lean or muscle mass (or size) following exercise [29,34,35,38,43,46,50,51]. Only Lima et al. found a significant increase in fat-free mass (kg and %) and lean body mass when compared to control [46].

In contrast, Painter et al. found an increased lean mass in both the exercise and control groups with no difference in the changes during the 12 months [29]. Zhang et al. [50] reported no changes in fat-free mass and no change in rectus femoris muscle thickness was seen following 10 weeks of RT by Hernández Sánchez et al. [43]. In Tzvetanov et al., data on body composition were available only for the intervention group [34]. Mean lean mass increased from 60.8 kg at baseline to 62.0 kg at 6 months and 63.1 kg at 12 months, although this was not significant. No significant change in lean body mass (assessed using skinfolds) was found in Riess et al. between groups (exercise 0.9 ± 3.64 kg vs. usual care: −0.6 ± 2.69 kg) [35]. Lean mass % was reduced by 1.5 and 2.3% in both groups, although this was non-significant in Karelis et al. [38]. Dry lean weight increased slightly during the 15 months in the study by Knobbe et al. in the exercise group, but this change was not significantly different to control [51].

Overall, there was no evidence that exercise had a favorable effect on markers of lean or muscle mass (or size), with one of eight studies favoring the intervention (13% [95%CI: 0–35%], *p* = 1.00).

##### Markers of fat mass and adiposity

Seven studies reported changes in markers of fat mass and adiposity following exercise [29,34,38,44,46,50,51]. Tzvetanov et al. found the mean percent of fat decreased slightly throughout the course of 12 months (although no data were provided in the study) [34]. Lima et al. found a significant within-group reduction in waist circumference. They also reported a significant reduction in body fat% when compared to control [46]. Knobbe at al. found no significant between group difference in change in body fat% or body fat (kg) between exercise and control but showed a smaller increase in waist circumference in the exercise group at 15 months [51].

Painter et al. found an increased fat mass and body fat % in both the exercise and control groups with no difference in the changes during the 12 months [29]. Karelis et al. reported increases in fat mass % in both the exercise and control groups (4.8 and 6.4%, respectively) [38]. No changes in body fat % were found in Zhang et al. [50]. In their initial study, O’Brien et al. found no change in waist circumference [44], with no further changes reported at 6 months [45].

Overall, there was no evidence that exercise had a favorable effect on fat mass or markers of adiposity, with two of seven studies favoring the intervention (29% [95%CI: 4–71%], *p* = 1.00).

#### Cardiovascular responses

##### Pulse wave velocity (PWV)

Pulse wave velocity was measured in three studies [34,35,37]. Greenwood et al. found both AT (−2.2 [95%CI, 23.1–21.3] m/s) and RT (−2.6 [95%CI: 23.4–21.7 m/s]) reduced PWV after 12 weeks [37]. In a 9-month follow-up of the ExeRT cohort, O’Connor et al. found, compared to usual care, RT significantly reduced PWV by −1.30 m/s (95%CI −2.44 to −0.17), while no significant difference was seen following AT (−1.05 m/s (95%CI: −2.11 to 0.017) [41]. In Tzvetanov et al., serial measures of central PWV were available only for the intervention group. The mean central PWV decreased substantially from 9.4 ± 6.3 m/s at baseline to 7.7 ± 1.7 m/s at 12 months [34]. The authors also measured carotid intima-media thickness and found a non-significant decrease from 0.64 ± 0.2 mm at baseline to 0.60 ± 0.0 mm at 12 months in the exercise group. Riess et al. used arterial pulse waveform analysis to measure artery compliance [35]. They found small artery compliance and large artery compliance were not different between groups.

##### Cardiovascular disease risk assessment

Two studies reported changes in CVD risk. No differences between the exercise and usual care groups in the Framingham CVD risk score [35] or 10-year coronary heart disease risk were reported [30].

#### Strength, physical function, and exercise capacity

##### Six-minute walking test (6MWT)

Five studies reported changes in 6MWT distance following exercise [40,42–44,50]. Results from these studies were not meta-analyzed due to high statistical heterogeneity. In Hernández Sánchez et al., a significant improvement was observed in the training group (9% vs. 1% in the control) [43]. Kumar et al. showed that after 12 weeks, while 6MWT improved in both control (+147.4 m) and intervention (+255.0 m) groups, the difference was greater in those undergoing exercise [42]. In Zhang et al., patients in the intervention group had longer walking distances (+10.4 m) than patients in the control group (−14.6 m) (*p* = .008), although the difference between groups was modest [50].

In contrast, a pilot study by O’Brien et al. found exercise did not increase 6MWT performance compared to the control group [44]. Onofre et al. found a significant reduction in 6MWT in both groups at discharge, and an exercise protocol commencing immediately after transplantation did not increase the distance walked [40].

Overall, there was no evidence that exercise had a favorable effect (defined as an increase >65 m, an estimate of the tests minimal detectable change [57], on 6MWT performance, with two of five studies favoring the intervention (40% [95%CI: 5–85%], *p* = 1.00).

##### Strength

Eight studies reported changes in strength following exercise [29,32,35,37,38,40,42,51]. In the Exercise in Renal Transplant (ExeRT) study, Greenwood et al. found isometric knee extensor strength was increased following RT but not AT, when compared to usual care [37]. Riess et al. reported leg press and leg extension 1RMs were significantly greater in the exercising group vs. usual care; however, leg curl 1RM did not change [35]. Painter et al. found the change in quadriceps peak torque during the 12 months was greater in the exercise compared with the usual care group [29]. Similarly, Kumar at al. found muscle strength assessed by isometric quadriceps strength was significantly improved compared to the control group [42]. A significant increase in muscle strength index (+29.2%) was reported by Karelis et al. using a composite score of lower-body strength assessed using leg press machine and upper-body strength via a seated chest press machine [38]. Knobbe et al. reported that change in overall muscle strength, assessed by the mean *z*-score of four muscle groups was significantly higher in exercise versus control at 15 months [51].

In Onofre et al., no differences in lower limb isometric contraction strength at discharge between exercising and control groups [40]. While no change in upper isometric contraction limb strength at discharge in the control group, a significant reduction in strength was seen in the exercising group. In contrast, Hernández Sánchez et al. found no change in concentric isokinetic knee extension and flexion torques of the dominant and non-dominant legs measured at 60°/s [43]. Korabiewska et al. reported no significant change in upper extremities (handgrip) muscle strength following 6 months of rehabilitation [32].

Overall, there was no evidence that exercise had a favorable effect on lower limb strength, with six of eight studies favoring the intervention (75% [95%CI: 35–97%], *p* = .289).

#### Patient-reported outcome measures (PROMs)

##### Short-form-36 item (SF-36)

Seven studies reported changes in SF-36 scores following exercise [29,34,35,37,43,50,51]. Results were not meta-analyzed due to high statistical heterogeneity. Tzvetanov et al. showed mean SF-36 score at 6 months was significantly higher in the intervention group compared with the control group (583 ± 13 vs. 436 ± 22, *p* = .008), although data were not presented for 12 months [34]. In Riess et al., the exercise group had a significant improvement in social functioning, mental composite score, and overall QoL scores compared with usual care [35]. Hernández Sánchez et al. found resistance exercise significantly increased some SF-36 domains (role-physical and vitality) but not all [43].

Conversely, Greenwood et al. found neither RT or AT had a significant effect on the physical composite or the mental composite score of the SF-36 questionnaire [37]. In Painter et al., no changes in composite or subscale scores were observed between the groups [29]. No changes in any of the domains of the SF-36 were also reported by Zhang et al. [50]. Knobbe et al. found no significant differences in the change in health-related QoL subdomain physical functioning assessed by the SF-36 between exercise and control at 15 months and similarly in the SF-36 physical and mental composite scores [51].

Overall, there was no evidence that exercise had a favorable effect on SF-36 scores, with three of seven studies favoring the intervention (57% [95%CI: 18–90%], *p* = 1.00).

##### Other PROMs

Several other PROMs were reported across studies with mixed findings. Karelis et al. found a significant increase in the WHO-5 well-being score after exercise [38], while Hernández Sánchez et al. showed no significant change in any domains of the Kidney Disease and Quality of Life (KDQOL-SF) [43]. Kastelz et al. found 12 months of exercise resulted in a significant increase in Global Physical Health and Global Mental Health using the PROMIS 10, and Physical Function from the PROMIS 29 [47]. No changes in domains of anxiety, depression, fatigue, pain, or sleep were seen.

In an assessment of self-reported functional status, Greenwood et al. revealed a significant mean difference in Duke Activity Status Index (DASI) score between RT and usual care groups at 12 weeks (8.8 ± 3.4 *p* = .01, 95%CI 2.0–15.6) [37]. There was no significant difference in DASI score between the AT group and usual care.

Fatigue was reduced across all domains of the 20-item multidimensional fatigue inventory (MFI-20) scale by Zhang et al. [50]. Similarly, fatigue using a Fatigue Severity Score was significantly reduced following exercise in Kumar et al. [42].

Zhang et al. reported that patients in the intervention group had lower Self-Rating Anxiety Scale (SAS) and Self-Rating Depression Scale (SDS) scores for depression and anxiety [50]. In Pooranfar et al., compared to control, in the exercise training group, sleep quality was improved by 27% and the sleep quantity was increased by 30 min [36].

#### Clinical markers

##### Creatinine

Seven studies reported changes in creatinine following exercise but were not meta-analyzed due to high statistical heterogeneity [29,31,32,34,43,46,50]. In Onofre et al., while both groups showed a significant reduction in serum creatinine at discharge, 2.1 (±1.3) and 2.7 (±1.1) mg/dL respectively for control and exercise, there were no statistical differences between groups [40]. Tzvetanov et al. found no significant difference after 12 months of exercise between the intervention and control group (1.41 ± 0.51 vs. 1.61 ± 0.54 mg/dL) [34]. No changes were also reported in three other studies [29,32,50]. Lima et al. found reduced serum creatinine after 12 weeks of combined training, compared to a small increase in the control [46]. No between-group analysis limits further statistical interpretation.

Overall, there was no evidence that exercise had a favorable effect on creatinine, with one of seven studies favoring the intervention (14% [95%CI: 0–58%], *p* = 1.00).

##### Estimated glomerular filtration rate (eGFR)

Like creatinine, overall, no changes were seen in eGFR. Greenwood et al. found no change in eGFR in both AT or RT groups [37]. Tzvetanov et al. reported a trend for an improvement in the intervention group compared with the control for eGFR (55.5 ± 18.6 vs. 38.8 ± 18.9 mL/min/1.73 m^2^), although this was non-significant [34]. In Lima et al., those in the exercise group had an increase in eGFR, compared to a reduction in the control group [46]. No between-group analysis limits further statistical interpretation.

##### Inflammatory markers

Only two studies reported on the effect of exercise on inflammatory markers [37,49]. Greenwood et al. found that compared to usual care, the AT or RT intervention had no significant effect on high-sensitivity C-reactive protein, TNF-a, TNFR-1, TNFR-2, fetuin A, or interleukin (IL) 6 values [37]. Hemmati et al. reported following 10 weeks of exercise, decreased levels of TNF-α and no significant differences in the IL-35, IL-31, and IL-4 levels [49]. Gene expression profiles showed significantly increased expression of T-bet and no changes in the GATA-3, RORYt, and FOXP3 levels.

##### Hemoglobin A1C

No change in A1C (%) was seen by Karelis et al. [38] while following 6 months of exercise, there was no change in glycated hemoglobin between groups reported by Michou et al. [48].

##### Total cholesterol

In Pooranfar et al., total cholesterol was significantly decreased after 10 weeks of exercise training in the exercise group compared to the control group [36]. Conversely, no change in total cholesterol was reported in Karelis et al. [38].

### Meta-analysis synthesis

#### Body mass and body composition

##### Body mass

**Figure 4.**
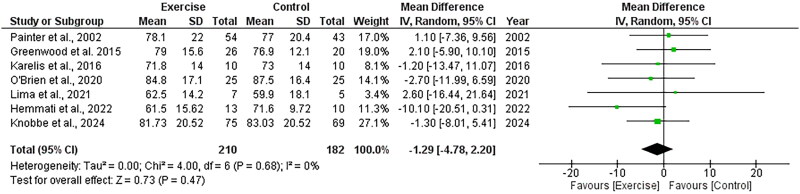
Forest plot for body mass. 95%CI: confidence interval (95%); SD: standard deviation.

Seven RCTs, including 392 participants, explored the effect of exercise on body mass and included data appropriate for meta-analysis. There was no significant change in body mass following exercise compared to control, with an overall MD of −1.29 kg (95%CI: −4.78 to +2.20, *Z* = 0.73, *p* = .47, Figure 4). Statistical heterogeneity was low (*I*^2^ = 0%).

##### Body mass index (BMI)

**Figure 5.**
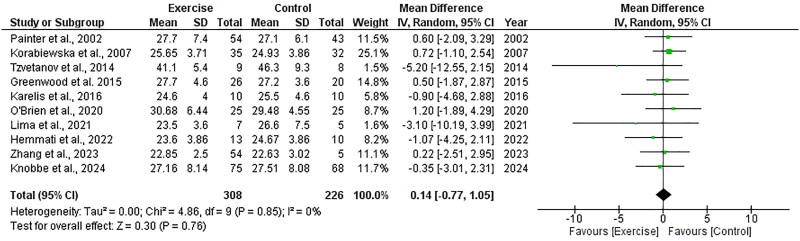
Forest plot for body mass index. 95%CI: confidence interval (95%); SD: standard deviation.

Ten RCTs, including 534 participants, explored the effect of exercise on BMI and included data appropriate for meta-analysis. There was no significant change in BMI following exercise compared to control, with an overall MD of +0.14 kg/m^2^ (95%CI: −0.77 to +1.05, *Z* = 0.30, *p* = .76, Figure 5). Statistical heterogeneity was low (*I*^2^ = 0%).

##### Bone mineral density (BMD)

**Figure 6.**
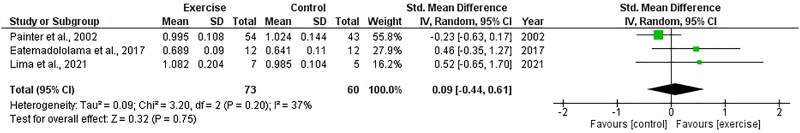
Forest plot for bone mineral density. 95%CI: confidence interval (95%); SD: standard deviation.

Three RCTs, including 133 participants, explored the effect of exercise on BMD and included data appropriate for meta-analysis. There was no significant change in BMD following exercise compared to control, with an overall SMD of 0.09 (95%CI: −0.44 to +0.61, *Z* = 0.32, *p* = .75, Figure 6). Translation of the SMD (using a SD of 0.204) led to an estimated change in BMD of +0.02 g/cm^2^ (95%CI: −0.09 to +0.12). Statistical heterogeneity was low (*I*^2^ = 37%).

#### Cardiovascular responses

##### Maximum heart rate

**Figure 7.**
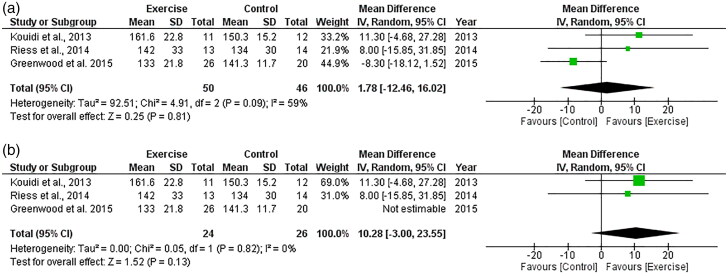
Forest plot for maximum heart rate. 95%CI: confidence interval (95%); SD: standard deviation.

Three RCTs, including 96 participants, explored the effect of exercise on maximum heart rate. Heterogeneity was high (*I*^2^ = 59%, Figure 7(A)) so Greenwood et al. was removed as outlined in the review methodology. As such, two studies included data appropriate for meta-analysis. There was no significant change in maximum heart rate following exercise compared to control, with an overall MD of +10.28 bpm (95%CI: −3.00 to +23.55, *Z* = 1.52, *p* = .13, Figure 7(B)). Statistical heterogeneity was low (*I*^2^ = 0%).

##### Resting heart rate

**Figure 8.**
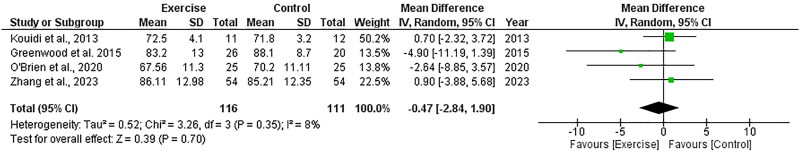
Forest plot for resting heart rate. 95%CI: confidence interval (95%); SD: standard deviation.

Four RCTs, including 227 participants, explored the effect of exercise on resting HR and included data appropriate for meta-analysis. There was no significant change in resting HR following exercise compared to control, with an overall MD of −0.47 bpm (95%CI: −2.84 to +1.90, *Z* = 0.39, *p* = .70, Figure 8). Statistical heterogeneity was low (*I*^2^ = 8%).

A 6-month follow-on study by O’Brien et al. reported no changes in HR [45].

##### Systolic blood pressure

**Figure 9.**
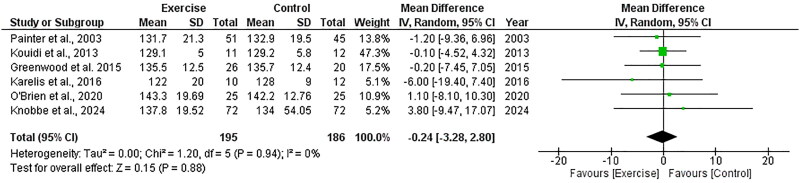
Forest plot for systolic blood pressure. 95%CI: confidence interval (95%); SD: standard deviation.

Six RCTs, including 381 participants, explored the effect of exercise on systolic blood pressure and included data appropriate for meta-analysis. There was no significant change in systolic blood pressure following exercise compared to control, with an overall MD of −0.24 mmHg (95%CI: −3.28 to +2.80, *Z* = 0.15, *p* = .88, Figure 9). Statistical heterogeneity was low (*I*^2^ = 0%).

A 6-month follow-on study by O’Brien et al. reported no changes in systolic blood pressure [45].

##### Diastolic blood pressure

**Figure 10.**
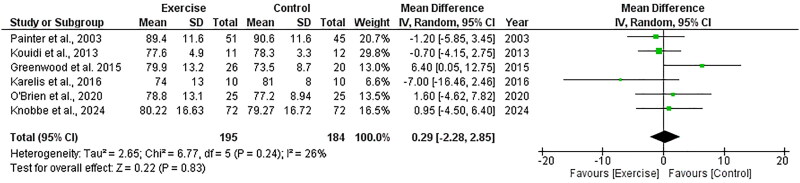
Forest plot for diastolic blood pressure. 95%CI: confidence interval (95%); SD: standard deviation.

Six RCTs, including 379 participants, explored the effect of exercise on diastolic blood pressure and included data appropriate for meta-analysis. There was no significant change in diastolic blood pressure following exercise compared to control, with an overall MD of +0.29 mmHg (95%CI: −2.28 to +2.85, *Z* = 0.22, *p* = .83, Figure 10). Statistical heterogeneity was low (*I*^2^ = 26%).

A 6-month follow-on study by O’Brien et al. reported no changes in diastolic blood pressure [45].

#### Strength, physical function, and exercise capacity

##### V̇O_2peak_ (cardiorespiratory fitness)

**Figure 11.**
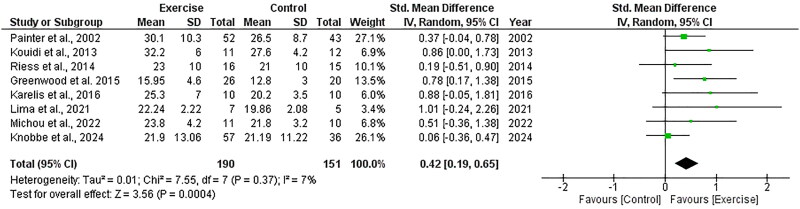
Forest plot for V̇O_2peak_. 95%CI: confidence interval (95%); SD: standard deviation.

Eight RCTs, including 341 participants, explored the effect of exercise on V̇O_2peak_ and included data appropriate for meta-analysis. There was a significant increase in V̇O_2peak_ following exercise compared to control, with an overall SMD of +0.42 (95%CI: +0.19 to +0.65, *Z* = 3.56, *p* = .0004, Figure 11). Translation of the SMD (using a SD of 9.21) led to an estimated increase in V̇O_2peak_ of +3.87 mL/min/kg (95%CI: +1.75 to +5.99). Statistical heterogeneity was low (*I*^2^ = 7%).

##### Sit-to-stand-60 s test (STS-60)

**Figure 12.**
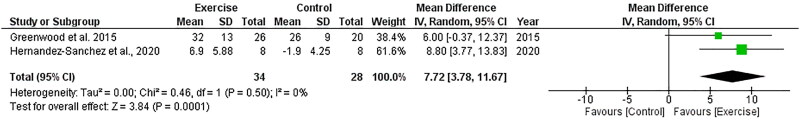
Forest plot for STS-60. Due to baseline imbalances, for Hernández Sánchez et al., the change from baseline data was used with 95%CI converted to SD. 95%CI: confidence interval (95%); SD: standard deviation.

Two RCTs, including 62 participants, explored the effect of exercise on the STS-60 and included data appropriate for meta-analysis. There was a significant increase in STS-60 performance following exercise compared to control, with an overall MD of +7.72 reps (95%CI: +3.78 to +11.67, *Z* = 3.84, *p* = .0001, Figure 12). Statistical heterogeneity was low (*I*^2^ = 0%).

##### Handgrip strength

**Figure 13.**
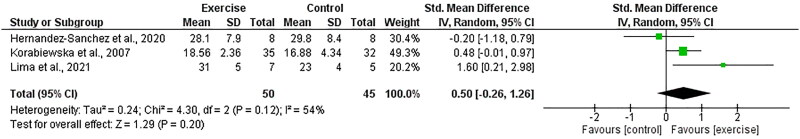
Forest plot for handgrip strength. 95%CI: confidence interval (95%); SD: standard deviation.

Three RCTs, including 95 participants, explored the effect of exercise on handgrip strength and included data appropriate for meta-analysis. There was no significant change in handgrip strength following exercise compared to control, with an overall SMD of +0.50 (95%CI: −0.26 to +1.26, *Z* = 1.29, *p* = .20, Figure 13). Translation of the SMD (using a SD of 7.9) led to an estimated change in handgrip strength of +3.95 kg (95%CI: −2.05 to +9.95). Statistical heterogeneity was moderate (*I*^2^ = 54%).

#### Clinical markers

##### Glucose

**Figure 14.**
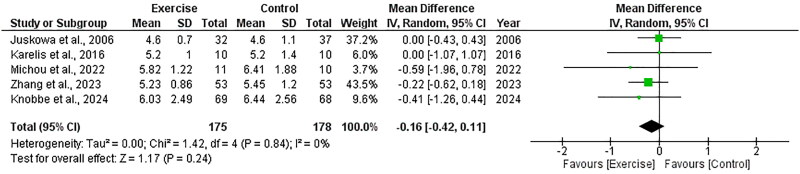
Forest plot for glucose. 95%CI: confidence interval (95%); SD: standard deviation.

Five RCTs, including 353 participants, explored the effect of exercise on glucose and included data appropriate for meta-analysis. There was no significant change in glucose following exercise compared to control, with an overall MD of −0.16 mmol/L (95%CI: −0.42 to +0.11, *Z* = 1.17, *p* = .24, Figure 14). Statistical heterogeneity was low (*I*^2^ = 0%).

##### Hemoglobin

**Figure 15.**
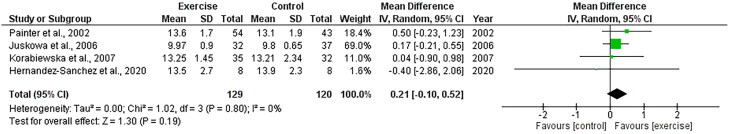
Forest plot for hemoglobin. 95%CI: confidence interval (95%); SD: standard deviation.

g 249 participants, explored the effect of exercise on hemoglobin and included data appropriate for meta-analysis. There was no significant change in hemoglobin following exercise compared to control, with an overall MD of +0.21 g/dL (95%CI: −0.10 to +0.52, *Z* = 1.30, *p* = .19, Figure 15). Statistical heterogeneity was low (*I*^2^ = 0%).

##### HDL

**Figure 16.**
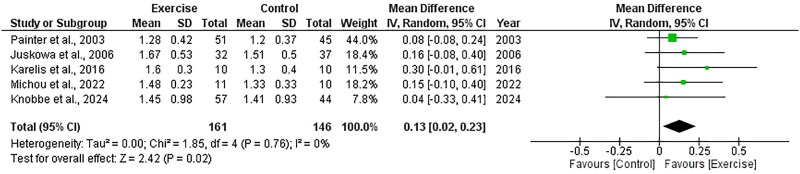
Forest plot for HDL. 95%CI: confidence interval (95%); SD: standard deviation.

Five RCTs, including 307 participants, explored the effect of exercise on HDL and included data appropriate for meta-analysis. There was a significant increase in HDL following exercise compared to control, with an overall MD of +0.13 mmol/L (95%CI: +0.02 to +0.23, *Z* = 2.42, *p* = .02, Figure 16). Statistical heterogeneity was low (*I*^2^ = 0%).

##### LDL

**Figure 17.**
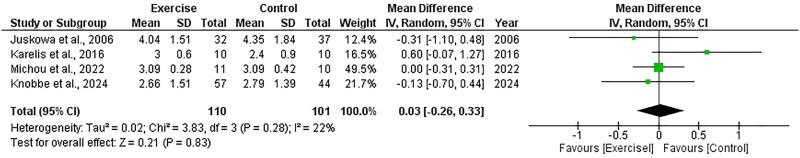
Forest plot for LDL. 95%CI: confidence interval (95%); SD: standard deviation.

Four RCTs, including 211 participants, explored the effect of exercise on LDL and included data appropriate for meta-analysis. There was no significant change in LDL following exercise compared to control, with an overall MD of +0.03 mmol/L (95%CI: −0.26 to +0.33, *Z* = 0.21, *p* = .83, Figure 17). Statistical heterogeneity was low (*I*^2^ = 22%).

##### IL-6

**Figure 18.**
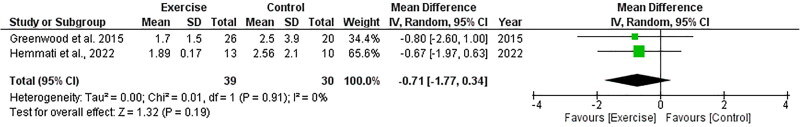
Forest plot for IL-6. 95%CI: confidence interval (95%); SD: standard deviation.

Two RCTs, including 69 participants, explored the effect of exercise on IL-6 and included data appropriate for meta-analysis. There was no significant change in IL-6 following exercise compared to control, with an overall MD of −0.71 pg/mL (95%CI: −1.77 to +0.34, *Z* = 1.32, *p* = .19, Figure 18). Statistical heterogeneity was low (*I*^2^ = 0%).

##### Triglycerides

**Figure 19.**
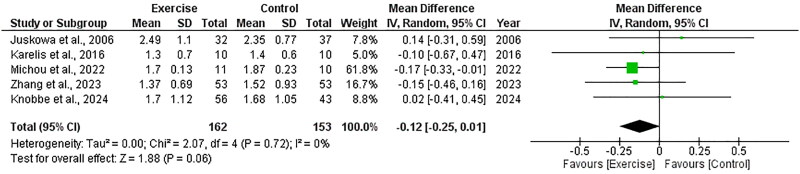
Forest plot for triglycerides. 95%CI: confidence interval (95%); SD: standard deviation.

Five RCTs, including 315 participants, explored the effect of exercise on triglycerides and included data appropriate for meta-analysis. There was no significant change in triglycerides following exercise compared to control, with an overall MD of −0.12 mmol/L (95%CI: −0.25 to 0.01, *Z* = 1.88, *p* = .06, Figure 19). Statistical heterogeneity was low (*I*^2^ = 0%).

## Discussion

Increasing physical activity through appropriate intervention should be a key component of post-transplant rehabilitation and a healthy lifestyle for KTRs, especially for the prevention and reduction of highly prevalent CVD within this population. However, evidence for the benefits of exercise in KTRs remains extremely limited. This review examined 23 RCTs with exercise interventions that varied in duration, frequency, and type, involving a diverse group of participants. The additional seven RCTs identified since our previous systematic review did not significantly alter the conclusions of the previous findings [24]. This updated review found favorable effects on CRF (V̇O_2peak_), physical function (STS-60), and HDL. However, no significant changes were observed in body mass, blood pressure, or other markers of dyslipidemia and glucose regulation. Some studies reported improvements in endothelial function, QoL, strength, and body composition. Despite these findings, the conclusions are constrained by small sample sizes, RoB, and statistical heterogeneity.

KTRs often exhibit diminished exercise capacity and low muscle function. Findings showed that exercise improved CRF. Peak V̇O_2_ (V̇O_2peak_) is considered the gold standard measure of cardiorespiratory function, and low V̇O_2peak_ consistently predicts mortality. In individuals awaiting a kidney transplant, it can also predict future cardiac events [58]. Improvements in CRF were typically observed after programs that included an aerobic component, while increases in muscle strength of upper and lower body muscle groups were achieved through the addition of RT. Changes in muscle strength are likely due to improvements in muscle mass and/or metabolic functioning, as increases in lean tissue were also observed [29,34]. An encouraging recent study by Knobbe et al. showed that V̇O_2peak_ improved after a 3-month supervised exercise intervention and this improvement was maintained at 15 months following a further 12 months of minimally resource intensive lifestyle counseling [51].

Dyslipidemia is common in KTRs [59], potentially partly due to immunosuppressive medication [60], and increases the risk of CVD. Apart from HDL, no significant effects on lipoproteins were observed. Exercise, when of sufficient intensity, is widely recognized to raise HDL levels [61], and the findings of this review align with the Pei et al. meta-analysis in patients with non-dialysis CKD [62]. Given that low HDL levels are associated with graft failure in KTRs, exercise may be a beneficial strategy to increase HDL and potentially maintain graft function [63]. The reasons for the lack of effect on many traditional CVD risk factors are unclear and likely confounded by the multiple pathological factors contributing to high CVD risk in these patients. It is important to note that many of the interventions were of short duration, which may not be sufficient to have an effect on these factors.

The majority of studies included in this review recruited patients with an established transplant, but some studies recruited participants immediately post-transplant. Onofre et al. implemented a daily supervised physiotherapy program that included both walking and RT immediately post-transplantation [40], while Juskowa et al. recruited patients 2–3 days post-transplant and had them participate in daily supervised and unsupervised strengthening exercises [31]. Neither study reported significant benefits from exercise. In Onofre et al., intensive physiotherapy did not mitigate the reductions in exercise capacity or peripheral muscle strength compared to standard care, which included simple mobility encouragement. Conversely, Zhang et al. reported significant improvements in fatigue, motivation, 30 s chair stands, and 6MWT result after an immediate post-transplant program of exercise that was continued remotely after discharge [50]. Therefore, there is conflicting evidence of the benefits of immediate post-transplant exercise programs compared to usual care.

Our updated methodology to explore statistical heterogeneity more thoroughly resulted in some outcomes previously meta-analyzed being deemed unsuitable in this updated review. For example, the 6MWT (previously statistically significant with an *I*^2^ of 77%), with the addition of one further study, remained highly heterogeneous and revealed no indication of a positive result through our new narrative synthesis. While the review of Zhang et al. concludes positive effects of exercise training on kidney function [28], our data do not support this due to the high statistical heterogeneity. Decreased kidney function is a risk factor for CVD [64]. Increased eGFR (through improved creatinine clearance) is expected post-transplantation and is used as a surrogate marker for allograft survival [65]. Although evidence for the additive effects of exercise on kidney function in KTRs is mixed, in non-dialysis CKD, meta-analyses have found favorable effects, likely through reductions in blood pressure and BMI [66]. As well as the variation between studies, the variation in transplant vintage and the changes in kidney function that occur naturally due to transplantation may have confounded exercise effects. Complex physiological and metabolic changes occur post-transplantation, without the presence of any significant intervention [67–69], which could influence studies that recruit within the first year after surgery.

There are no studies included within the review which examine the use of digital health interventions or virtual reality exercise as a method of engagement and improving adherence to physical activity and exercise. Virtual reality training has been shown to be promising in patients receiving hemodialysis for reductions in anxiety and depression and improvements in self-efficacy [70]. Digital health interventions such as Kidney BEAM have been shown to improve mental health-related QoL, patient activation, and physical function in patients with CKD (including KTRs) [71].

Several limitations of the methodology of the original review were addressed in this updated review. Random effects meta-analyses were deemed most appropriate as the variation between interventions was large. A rigorous process was followed to determine whether meta-analysis was appropriate by exploring the sources of heterogeneity following more closely the advice in the Cochrane Handbook [52–54]. And finally, a more detailed narrative synthesis of results unsuitable for meta-analysis with a direction of effect was included. Nonetheless, this review has some limitations. Although the search was restricted to only RCTs, which reduced some bias, the trial designs were inconsistent. Additionally, certain study designs and methods, including randomization, were not clearly described. While the review aimed to capture as many outcomes as possible, the numerous and heterogeneous quality of the reported outcomes limited data synthesis. Overall, sample sizes were small, with the exception of the recent study by Knobbe at al. (overall *n* = 221). The review revealed that basic training principles, such as exercise modality, intensity, and frequency, were poorly described, making replication of these interventions difficult. Most studies included interventions of relatively short duration, preventing conclusions about long-term effects. None of the studies reported the impact of exercise training on ‘hard’ clinical outcomes such as mortality or graft function, often relying on surrogate markers instead.

## Conclusions

The results of the meta-analysis demonstrate a mixed impact of physical activity and/or exercise training interventions on outcomes. There was a positive effect on CRF (V̇O_2peak_), physical function (STS-60), and some markers of dyslipidemia. Given the links between CRF and CVD risk, these data are encouraging to suggest interventions may have a positive impact on cardio-metabolic health. However, there were no effects on factors such as body mass and composition or glycemic control. The studies were highly heterogeneous in sample size, duration, intervention content, outcome measure choice, and collection method. Exercise protocols were poorly reported, leading to difficulties in study replication and clinical implementation, which is synonymous with the findings in a review of exercise trials in solid organ transplant recipients [72]. Further long-term, thoroughly reported, large sample RCTs are needed to fully understand the effects of increasing physical activity levels and exercise in KTRs.

## Supplementary Material

Supplemental Material

## Data Availability

Data collection forms, data extracted from included studies, and data used for all analyses can be requested from the corresponding author.

## References

[1] Tonelli M, Wiebe N, Knoll G, et al. Systematic review: kidney transplantation compared with dialysis in clinically relevant outcomes. Am J Transplant. 2011;11(10):2093–2109. doi: 10.1111/j.1600-6143.2011.03686.x. 21883901

[2] UK Renal Registry. UK Renal Registry 26th Annual Report – data to 31/12/2022. Bristol (UK): UK Renal Registry; 2024.

[3] Burton H, Iyamu Perisanidou L, Steenkamp R, et al. Causes of renal allograft failure in the UK: trends in UK Renal Registry and National Health Service Blood and Transplant data from 2000 to 2013. Nephrol Dial Transplant. 2019;34(2):355–364. doi: 10.1093/ndt/gfy168. 29982787

[4] Devine PA, Courtney AE, Maxwell AP. Cardiovascular risk in renal transplant recipients. J Nephrol. 2019;32(3):389–399. [published Online First: 2018/11/07] doi: 10.1007/s40620-018-0549-4. 30406606 PMC6482292

[5] Baker LA, March DS, Wilkinson TJ, et al. Clinical practice guideline exercise and lifestyle in chronic kidney disease. BMC Nephrol. 2022;23(1):75. doi: 10.1186/s12882-021-02618-1. 35193515 PMC8862368

[6] Nielens H, Lejeune TM, Lalaoui A, et al. Increase of physical activity level after successful renal transplantation: a 5 year follow-up study. Nephrol Dial Transplant. 2001;16(1):134–140. [published Online First: 2001/02/24] doi: 10.1093/ndt/16.1.134. 11209007

[7] Dontje ML, de Greef MH, Krijnen WP, et al. Longitudinal measurement of physical activity following kidney transplantation. Clin Transplant. 2014;28(4):394–402. [published Online First: 2014/03/19] doi: 10.1111/ctr.12325. 24635476

[8] Wilkinson T, Clarke A, Nixon D, et al. Prevalence and correlates of physical activity across kidney disease stages: an observational multicentre study. Nephrol Dial Transplant. 2021;36(4):641–649. doi: 10.1093/ndt/gfz235. 31725147

[9] van Adrichem EJ, van de Zande SC, Dekker R, et al. Perceived barriers to and facilitators of physical activity in recipients of solid organ transplantation, a qualitative study. PLOS One. 2016;11(9):e0162725. doi: 10.1371/journal.pone.0162725. 27622291 PMC5021267

[10] Gordon EJ, Prohaska TR, Gallant M, et al. Self-care strategies and barriers among kidney transplant recipients: a qualitative study. Chronic Illn. 2009;5(2):75–91. doi: 10.1177/1742395309103558. 19474231 PMC3540789

[11] Zelle DM, Klaassen G, van Adrichem E, et al. Physical inactivity: a risk factor and target for intervention in renal care. Nat Rev Nephrol. 2017;13(3):152–168. [published Online First: 20170131] doi: 10.1038/nrneph.2016.187. 28138130

[12] Painter P. Exercise following organ transplantation: a critical part of the routine post transplant care. Ann Transplant. 2005;10(4):28–30. 17037085

[13] Zelle DM, Corpeleijn E, Klaassen G, et al. Fear of movement and low self-efficacy are important barriers in physical activity after renal transplantation. PLOS One. 2016;11(2):e0147609. doi: 10.1371/journal.pone.0147609. 26844883 PMC4742485

[14] Billany RE, Bishop NC, Stevinson C, et al. Perceptions and experiences of high-intensity interval training in kidney transplant recipients: a big HIIT? Nephrol Nurs J. 2023;50(1):31–42. doi: 10.37526/1526-744X.2023.50.1.31. 36961072

[15] Billany RE, Smith AC, Stevinson C, et al. Perceived barriers and facilitators to exercise in kidney transplant recipients: a qualitative study. Health Expect. 2022;25(2):764–774. doi: 10.1111/hex.13423. 35014114 PMC8957725

[16] Sánchez ZV, Cashion AK, Cowan PA, et al. Perceived barriers and facilitators to physical activity in kidney transplant recipients. Prog Transplant. 2007;17(4):324–331. doi: 10.1177/152692480701700411. 18240699

[17] Zelle DM, Corpeleijn E, Stolk RP, et al. Low physical activity and risk of cardiovascular and all-cause mortality in renal transplant recipients. Clin J Am Soc Nephrol. 2011;6(4):898–905. doi: 10.2215/CJN.03340410. 21372213 PMC3069385

[18] Kang AW, Garber CE, Eaton CB, et al. Physical activity and cardiovascular risk among kidney transplant patients. Med Sci Sports Exerc. 2019;51(6):1154–1161. doi: 10.1249/MSS.0000000000001886. 30629045 PMC6522300

[19] MacKinnon HJ, Wilkinson TJ, Clarke AL, et al. The association of physical function and physical activity with all-cause mortality and adverse clinical outcomes in nondialysis chronic kidney disease: a systematic review. Ther Adv Chronic Dis. 2018;9(11):209–226. [published Online First: 20180704] doi: 10.1177/2040622318785575. 30364521 PMC6196637

[20] Castle EM, Billany RE, Lightfoot CJ, et al. Exercise as a therapeutic intervention in chronic kidney disease: are we nearly there yet? Curr Opin Nephrol Hypertens. 2023;32(6):502–508. doi: 10.1097/mnh.0000000000000923. 37622530 PMC10552838

[21] Battaglia Y, Baciga F, Bulighin F, et al. Physical activity and exercise in chronic kidney disease: consensus statements from the Physical Exercise Working Group of the Italian Society of Nephrology. J Nephrol. 2024;37(7):1735–1765. doi: 10.1007/s40620-024-02049-9. 39269600 PMC11519309

[22] Hutchinson GM, Cooper AM, Billany RE, et al. Effect of high intensity interval training and moderate intensity continuous training on lymphoid, myeloid and inflammatory cells in kidney transplant recipients. Exerc Immunol Rev. 2022;28:100–115. 35452395

[23] Liberati A, Altman DG, Tetzlaff J, et al. The PRISMA statement for reporting systematic reviews and meta-analyses of studies that evaluate health care interventions: explanation and elaboration. PLoS Med. 2009;6(7):e1000100. [published Online First: 20090721] doi: 10.1371/journal.pmed.1000100. 19621070 PMC2707010

[24] Wilkinson T, Bishop N, Billany R, et al. The effect of exercise training interventions in adult kidney transplant recipients: a systematic review and meta-analysis of randomised control trials. Phys Ther Rev. 2022;27(2):114–134. doi: 10.1080/10833196.2021.2002641.

[25] Mactier R, Hoenich N, Breen C. Renal Association Clinical Practice Guideline on haemodialysis. Nephron Clin Pract. 2011;118(Suppl. 1):c241–c286. [published Online First: 20110506] doi: 10.1159/000328072. 21555899

[26] Calella P, Hernández-Sánchez S, Garofalo C, et al. Exercise training in kidney transplant recipients: a systematic review. J Nephrol. 2019;32(4):567–579. [published Online First: 2019/01/17] doi: 10.1007/s40620-019-00583-5. 30649716

[27] Oguchi H, Tsujita M, Yazawa M, et al. The efficacy of exercise training in kidney transplant recipients: a meta-analysis and systematic review. Clin Exp Nephrol. 2019;23(2):275–284. [published Online First: 2018/09/01] doi: 10.1007/s10157-018-1633-8. 30168049

[28] Zhang D, Yu L, Xia B, et al. Systematic review and meta-analysis of the efficacy of exercise intervention in kidney transplant recipients. World J Urol. 2023;41(12):3449–3469. doi: 10.1007/s00345-023-04673-9. 37882807

[29] Painter P, Hector L, Ray K, et al. A randomized trial of exercise training after renal transplantation. Transplantation. 2002;74(1):42–48. doi: 10.1097/00007890-200207150-00008. 12134097

[30] Painter P, Hector L, Ray K, et al. Effects of exercise training on coronary heart disease risk factors in renal transplant recipients. Am J Kidney Dis. 2003;42(2):362–369. doi: 10.1016/s0272-6386(03)00673-5. 12900820

[31] Juskowa J, Lewandowska M, Bartłomiejczyk I, et al. Physical rehabilitation and risk of atherosclerosis after successful kidney transplantation. Transplant Proc. 2006;38(1):157–160. doi: 10.1016/j.transproceed.2005.12.077. 16504691

[32] Korabiewska L, Lewandowska M, Juskowa J, et al. Need for rehabilitation in renal replacement therapy involving allogeneic kidney transplantation. Transplant Proc. 2007;39(9):2776–2777. doi: 10.1016/j.transproceed.2007.08.082. 18021985

[33] Kouidi E, Vergoulas G, Anifanti M, et al. A randomized controlled trial of exercise training on cardiovascular and autonomic function among renal transplant recipients. Nephrol Dial Transplant. 2013;28(5):1294–1305. [published Online First: 20121104] doi: 10.1093/ndt/gfs455. 23129823

[34] Tzvetanov I, West-Thielke P, D’Amico G, et al. A novel and personalized rehabilitation program for obese kidney transplant recipients. Transplant Proc. 2014;46(10):3431–3437. doi: 10.1016/j.transproceed.2014.05.085. 25498067

[35] Riess KJ, Haykowsky M, Lawrance R, et al. Exercise training improves aerobic capacity, muscle strength, and quality of life in renal transplant recipients. Appl Physiol Nutr Metab. 2014;39(5):566–571. [published Online First: 20131128] doi: 10.1139/apnm-2013-0449. 24766239

[36] Pooranfar S, Shakoor E, Shafahi M, et al. The effect of exercise training on quality and quantity of sleep and lipid profile in renal transplant patients: a randomized clinical trial. Int J Organ Transplant Med. 2014;5(4):157–165. 25426284 PMC4243047

[37] Greenwood SA, Koufaki P, Mercer TH, et al. Aerobic or resistance training and pulse wave velocity in kidney transplant recipients: a 12-week pilot randomized controlled trial (the Exercise in Renal Transplant [ExeRT] trial). Am J Kidney Dis. 2015;66(4):689–698. [published Online First: 2015/07/26] doi: 10.1053/j.ajkd.2015.06.016. 26209542

[38] Karelis AD, Hébert MJ, Rabasa-Lhoret R, et al. Impact of resistance training on factors involved in the development of new-onset diabetes after transplantation in renal transplant recipients: an open randomized pilot study. Can J Diabetes. 2016;40(5):382–388. [published Online First: 2015/12/15] doi: 10.1016/j.jcjd.2015.08.014. 26656280

[39] Eatemadololama A, Karimi MT, Rahnama N, et al. Resistance exercise training restores bone mineral density in renal transplant recipients. Clin Cases Miner Bone Metab. 2017;14(2):157–160. [published Online First: 20171025] doi: 10.11138/ccmbm/2017.14.1.157. 29263725 PMC5726201

[40] Onofre T, Fiore Junior JF, Amorim CF, et al. Impact of an early physiotherapy program after kidney transplant during hospital stay: a randomized controlled trial. J Bras Nefrol. 2017;39(4):424–432. doi: 10.5935/0101-2800.20170075. 29319769

[41] O’Connor EM, Koufaki P, Mercer TH, et al. Long-term pulse wave velocity outcomes with aerobic and resistance training in kidney transplant recipients – a pilot randomised controlled trial. PLOS One. 2017;12(2):e0171063. [published Online First: 2017/02/06] doi: 10.1371/journal.pone.0171063. 28158243 PMC5291475

[42] Kumar TGS, Soundararajan P, Maiya AG, et al. Effects of graded exercise training on functional capacity, muscle strength, and fatigue after renal transplantation: a randomized controlled trial. Saudi J Kidney Dis Transpl. 2020;31(1):100–108. [published Online First: 2020/03/05] doi: 10.4103/1319-2442.279929. 32129202

[43] Hernández Sánchez S, Carrero JJ, Morales JS, et al. Effects of a resistance training program in kidney transplant recipients: a randomized controlled trial. Scand J Med Sci Sports. 2021;31(2):473–479. doi: 10.1111/sms.13853. 33038051

[44] O’Brien T, Russell CL, Tan A, et al. A pilot randomized controlled trial using SystemCHANGE™ approach to increase physical activity in older kidney transplant recipients. Prog Transplant. 2020;30(4):306–314. [published Online First: 20200910] doi: 10.1177/1526924820958148. 32912051 PMC7959697

[45] O’Brien T, Tan A, Rose K, et al. Maintenance phase of a physical activity intervention in older kidney transplant recipients: a 12-month follow-up. Geriatr Nurs. 2021;42(6):1541–1546. [published Online First: 20211103] doi: 10.1016/j.gerinurse.2021.08.019. 34741827 PMC8671339

[46] Lima PS, de Campos AS, de Faria Neto O, et al. Effects of combined resistance plus aerobic training on body composition, muscle strength, aerobic capacity, and renal function in kidney transplantation subjects. J Strength Cond Res. 2021;35(11):3243–3250. doi: 10.1519/jsc.0000000000003274. 31714457

[47] Kastelz A, Fernhall B, Wang E, et al. Personalized physical rehabilitation program and employment in kidney transplant recipients: a randomized trial. Transpl Int. 2021;34(6):1083–1092. doi: 10.1111/tri.13868. 33733479

[48] Michou V, Nikodimopoulou M, Deligiannis A, et al. Metabolic and functional effects of exercise training in diabetic kidney transplant recipients. World J Transplant. 2022;12(7):184–194. doi: 10.5500/wjt.v12.i7.184. 36051451 PMC9331407

[49] Hemmati N, Kazemi S, Jamshidian-Tehrani N, et al. Effects of exercise training on immunological factors in kidney transplant recipients; a randomized controlled trial. Res Sports Med. 2022;30(1):80–91. doi: 10.1080/15438627.2021.1906671. 33843376

[50] Zhang P, Liu S, Zhu X, et al. The effects of a physical exercise programme in Chinese kidney transplant recipients: a prospective randomised controlled trial. Clin Kidney J. 2023;16(8):1316–1329. doi: 10.1093/ckj/sfad065. 37529646 PMC10387397

[51] Knobbe TJ, Kremer D, Zelle DM, et al. Effect of an exercise intervention or combined exercise and diet intervention on health-related quality of life-physical functioning after kidney transplantation: the Active Care after Transplantation (ACT) multicentre randomised controlled trial. Lancet Healthy Longev. 2024;5(9):100622. [published Online First: 20240910] doi: 10.1016/j.lanhl.2024.07.005. 39270688

[52] Higgins J, Savović J, Page M, et al. Chapter 8: assessing risk of bias in a randomized trial. In: Higgins JPTSJ, Page MJ, Elbers RG, et al., editors. Cochrane handbook for systematic reviews of interventions. 2nd ed. Chichester (UK): John Wiley & Sons; 2019.

[53] Deeks J, Higgins J, Altman D, et al. Chapter 10: analysing data and undertaking meta-analyses. In: Higgins JPTSJ, Page MJ, Elbers RG, et al., editors. Cochrane handbook for systematic reviews of interventions. 2nd ed. Chichester (UK): John Wiley & Sons; 2019.

[54] Higgins J, Li T, Deeks J. Chapter 6: choosing effect measures and computing estimates of effect. In: Higgins JPTSJ, Page MJ, Elbers RG, et al., editors. Cochrane handbook for systematic reviews of interventions. 2nd ed. Chichester (UK): John Wiley & Sons; 2019.

[55] Tufanaru C, Munn Z, Stephenson M, et al. Fixed or random effects meta-analysis? Common methodological issues in systematic reviews of effectiveness. Int J Evid Based Healthc. 2015;13(3):196–207. 26355603 10.1097/XEB.0000000000000065

[56] Gallardo-Gómez D, Pedder H, Welton NJ, et al. Variability in meta-analysis estimates of continuous outcomes using different standardization and scale-specific re-expression methods. J Clin Epidemiol. 2024;165:111213. doi: 10.1016/j.jclinepi.2023.11.003. 37949198

[57] MacRae JM, Harasemiw O, Lightfoot CJ, et al. Measurement properties of performance-based measures to assess physical function in chronic kidney disease: recommendations from a COSMIN systematic review. Clin Kidney J. 2023;16(11):2108–2128. doi: 10.1093/ckj/sfad170. 37915888 PMC10616478

[58] Chakkera HA, Angadi SS, Heilman RL, et al. Cardiorespiratory fitness (peak oxygen uptake): safe and effective measure for cardiovascular screening before kidney transplant. J Am Heart Assoc. 2018;7(11):e008662. doi: 10.1161/jaha.118.008662. 29853444 PMC6015378

[59] Marubayashi S, Ohdan H, Tashiro H, et al. Studies on post-transplant dyslipidemia in kidney transplant patients. Hiroshima J Med Sci. 2005;54(2):39–45. 15991596

[60] Pham PT, Pham PM, Pham SV, et al. New onset diabetes after transplantation (NODAT): an overview. Diabetes Metab Syndr Obes. 2011;4:175–186. [published Online First: 20110509] doi: 10.2147/dmso.S19027. 21760734 PMC3131798

[61] Franczyk B, Gluba-Brzózka A, Ciałkowska-Rysz A, et al. The impact of aerobic exercise on HDL quantity and quality: a narrative review. Int J Mol Sci. 2023;24(5):4653. doi: 10.3390/ijms24054653. 36902082 PMC10003711

[62] Pei G, Tang Y, Tan L, et al. Aerobic exercise in adults with chronic kidney disease (CKD): a meta-analysis. Int Urol Nephrol. 2019;51(10):1787–1795. [published Online First: 2019/07/25] doi: 10.1007/s11255-019-02234-x. 31332699

[63] Annema W, Dikkers A, de Boer JF, et al. HDL cholesterol efflux predicts graft failure in renal transplant recipients. J Am Soc Nephrol. 2016;27(2):595–603. [published Online First: 20150828] doi: 10.1681/asn.2014090857. 26319244 PMC4731105

[64] Thompson S, James M, Wiebe N, et al. Cause of death in patients with reduced kidney function. J Am Soc Nephrol. 2015;26(10):2504–2511. doi: 10.1681/ASN.2014070714. 25733525 PMC4587695

[65] Hilbrands L, Budde K, Bellini MI, et al. Allograft function as endpoint for clinical trials in kidney transplantation. Transpl Int. 2022;35:10139. [published Online First: 20220520] doi: 10.3389/ti.2022.10139. 35669976 PMC9163811

[66] Zhang L, Wang Y, Xiong L, et al. Exercise therapy improves eGFR, and reduces blood pressure and BMI in non-dialysis CKD patients: evidence from a meta-analysis. BMC Nephrol. 2019;20(1):398. [published Online First: 20191029] doi: 10.1186/s12882-019-1586-5. 31664943 PMC6821004

[67] Ferris RL, Kittur DS, Wilasrusmee C, et al. Early hemodynamic changes after renal transplantation: determinants of low central venous pressure in the recipients and correlation with acute renal dysfunction. Med Sci Monit. 2003;9(2):CR61–CR66. 12601288

[68] Patti A, Neunhaeuserer D, Ortolan S, et al. A clinical evaluation of VO_2_ kinetics in kidney transplant recipients. Eur J Appl Physiol. 2021;121(7):2005–2013. doi: 10.1007/s00421-021-04672-x. 33811560 PMC8192378

[69] Tantisattamo E, Ho BT, Workeneh BT. Editorial: metabolic changes after kidney transplantation. Front Med. 2021;8:709644. [published Online First: 20210708] doi: 10.3389/fmed.2021.709644. PMC8297834 34307432

[70] Kang X, Zhang Y, Sun C, et al. Effectiveness of virtual reality training in improving outcomes for dialysis patients: systematic review and meta-analysis. J Med Internet Res. 2025;27:e58384. [published Online First: 20250108] doi: 10.2196/58384. 39773859 PMC11754980

[71] Greenwood SA, Young HML, Briggs J, et al. Evaluating the effect of a digital health intervention to enhance physical activity in people with chronic kidney disease (Kidney BEAM): a multicentre, randomised controlled trial in the UK. Lancet Digit Health. 2024;6(1):e23–e32. doi: 10.1016/S2589-7500(23)00204-2. 37968170

[72] Raje U, Saumur TM, Pesce de Souza F, et al. Quality of the reporting of exercise interventions in solid organ transplant recipients: a systematic review. McGill J Med. 2021;19(1). doi: 10.26443/mjm.v19i1.219.

