# Supplementary material for: Physical activity interventions in adult kidney transplant recipients: an updated systematic review and meta-analysis of randomized controlled trials
Source: Ren Fail. 2025 Mar 27;47(1):2480246. doi: 10.1080/0886022X.2025.2480246 (PMC11951324; doi:10.1080/0886022X.2025.2480246)
Supplement: Supplemental Material [file IRNF_A_2480246_SM0629.docx]

**Supplementary material 1. Full search strategies for updated review**

**MEDLINE via PubMed (March 2021-present day)**

**Final search details:**

(((("exercise"[MeSH Terms] OR "exercise"[All Fields]) OR ("exercise therapy"[MeSH Terms] OR ("exercise"[All Fields] AND "therapy"[All Fields]) OR "exercise therapy"[All Fields])) OR ("exercise"[MeSH Terms] OR "exercise"[All Fields] OR ("physical"[All Fields] AND "activity"[All Fields]) OR "physical activity"[All Fields])) AND ((("kidney transplantation"[MeSH Terms] OR ("kidney"[All Fields] AND "transplantation"[All Fields]) OR "kidney transplantation"[All Fields] OR ("renal"[All Fields] AND "transplantation"[All Fields]) OR "renal transplantation"[All Fields]) OR ("kidney transplantation"[MeSH Terms] OR ("kidney"[All Fields] AND "transplantation"[All Fields]) OR "kidney transplantation"[All Fields])) OR ("transplant recipients"[MeSH Terms] OR ("transplant"[All Fields] AND "recipients"[All Fields]) OR "transplant recipients"[All Fields]))) AND ("randomized controlled trial"[Publication Type] OR "randomized controlled trials as topic"[MeSH Terms] OR "randomized controlled trial"[All Fields] OR "randomised controlled trial"[All Fields])

Latest search date: **38**

Strategy structure and breakdown of search returns:

| **Search term** | **Items returned** |
| --- | --- |
| 1. Renal Transplantation | 21712 |
| 1. Kidney Transplantation (MESH) | 18988 |
| 1. Transplant Recipients (MESH) | 14641 |
| 1. 1 OR 2 OR 3   ((Renal Transplantation) OR Kidney Transplantation) OR Transplant Recipients | 29783 |
| 1. Exercise (MESH) | 111126 |
| 1. Exercise therapy (MESH) | 29603 |
| 1. Physical activity | 145598 |
| 1. 5 OR 6 OR 7   ((Exercise) OR Exercise therapy) OR Physical activity | 151983 |
| 1. 4 AND 8   ((((Exercise) OR Exercise therapy) OR Physical activity)) AND (((Renal Transplantation) OR Kidney Transplantation) OR Transplant Recipients) | 432 |
| 1. Randomi?ed controlled* trial (MESH) | 20339 |
| 1. 9 AND 10   ((((((Exercise) OR Exercise therapy) OR Physical activity)) AND (((Renal Transplantation) OR Kidney Transplantation) OR Transplant Recipients))) AND Randomized controlled trial | 38 |

Search retuned: 38 studies

32 studies were removed

| **Reason** | **Number excluded after abstract review** |
| --- | --- |
| Review paper | 2 |
| Dialysis | 2 |
| Non-dialysis CKD | 1 |
| Non-KD | 20 |
| Protocol paper | 5 |
| Non-exercise intervention | 4 |

| Remaining total based on initial review of abstract and full text sought | 6 |
| --- | --- |
| Removed after full text review |  |
| Added from hand-searching reference lists |  |
| Total left for review and analysis |  |

In the original review (Wilkinson et al. 2019), searches were conducted up until the 17^th^ March 2021. This updated review searched from 17th March 2021 to 7th May 2024.

**Cochrane Central Register of Controlled Trials (includes EMBASE, ICTRP)**

**Final search details:**

‘exercise therapy’ ‘physical activity’ ‘kidney transplantation’ ‘renal transplant recipients’ ‘randomized controlled trial’

Latest search date: 7/5/2024

Search retuned: #2 studies

#2 studies were removed

| **Reason** | **Number excluded after abstract review** |
| --- | --- |
| Duplicate | #1 |
| No exercise intervention | #1 |

Remaining total based on initial review of abstract and full text sought #0

In the original review (Wilkinson et al. 2019), searches were conducted up until the 17^th^ March 2021. This updated review searched from March 2021 to 7th May 2024.

**Other methods**

1 additional study was identified during another review by our group (Bishop et al., 2023)

**Supplementary material 2. Forest plots of excluded meta-analysis**

| *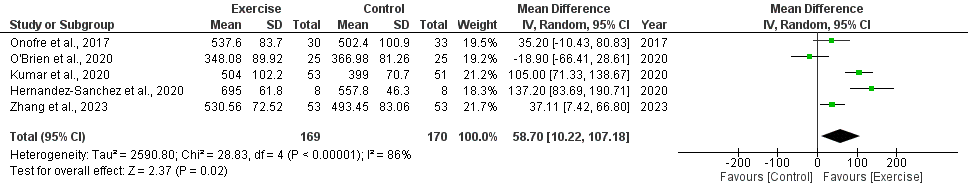* |
| --- |
| *Figure S1. Forest plot for six-minute walk test.*  *95% CI = confidence interval (95%); SD = Standard deviation* |

| *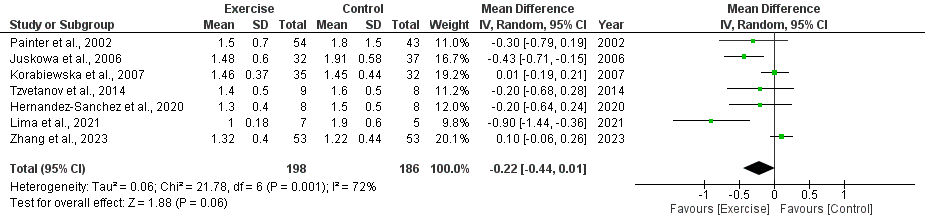* |
| --- |
| *Figure S2. Forest plot for creatinine.*  *95% CI = confidence interval (95%); SD = Standard deviation* |

| *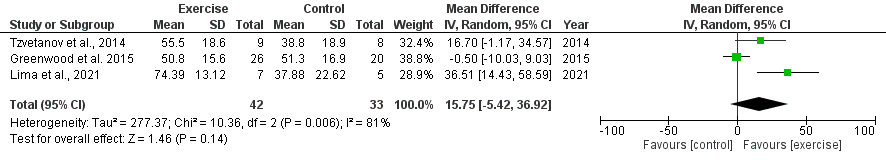* |
| --- |
| *Figure S3. Forest plot for eGFR.*  *95% CI = confidence interval (95%); SD = Standard deviation* |

| *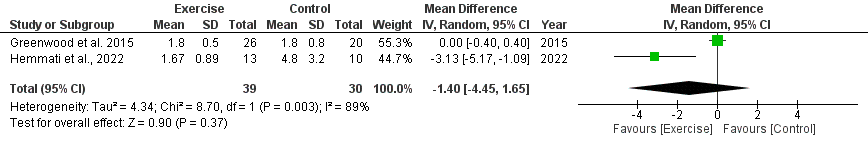* |
| --- |
| *Figure S4. Forest plot for TNF-a.*  *95% CI = confidence interval (95%); SD = Standard deviation* |
